# Biological findings from a newly developed photo-identification catalog for the critically endangered Rice’s whale (*Balaenoptera ricei*)

**DOI:** 10.1371/journal.pone.0331010

**Published:** 2025-09-08

**Authors:** Laura Aichinger Dias, Kevin P. Barry, Lance P. Garrison, Jenny Litz, Lynsey A. Wilcox Talbot, Ruth Y. Ewing, Anthony Martinez

**Affiliations:** 1 Cooperative Institute for Marine and Atmospheric Studies, University of Miami, Miami, Florida, United States of America; 2 Marine Mammal and Turtle Division, Southeast Fisheries Science Center, National Marine Fisheries Service, National Oceanic and Atmospheric Administration, Miami, Florida, United States of America; 3 Marine Mammal and Turtle Division, Southeast Fisheries Science Center, National Marine Fisheries Service, National Oceanic and Atmospheric Administration, Pascagoula, Mississippi, United States of America; 4 Marine Mammal and Turtle Division, Southeast Fisheries Science Center, National Marine Fisheries Service, National Oceanic and Atmospheric Administration, Lafayette, Louisiana, United States of America; Alaska Pacific University, UNITED STATES OF AMERICA

## Abstract

The Rice’s whale is among the world’s most endangered whales. It has a small population size, low genetic diversity, and is exposed to several anthropogenic threats. In this study, we compiled photographs taken from whale sightings during vessel-based research surveys conducted by the U.S. National Marine Fisheries Service, Southeast Fisheries Science Center between 2004 and 2019 and used photo-ID techniques to develop an identification catalog. Thirty-one whales were individually identified based on dorsal fin attributes and body marks. On the dorsal fin, lacerations, nicks and notches were the most commonly available attributes used for identification and matching. Cookiecutter shark bite scars were widely present on the body of the whales and also served for identification and matching. Of the 31 whales, 28 were sighted multiple times with time between sightings ranging from seven days to more than 15 years. Individual genotyping and sexing were available for 25 cataloged whales. Genotyping confirmed that whales identified via photographs were genetically unique and sexing resulted in 14 females and 11 males. Here we also present insights into rarely recorded presumed mother and calf pairs, with three female whales identified as presumed mothers. Finally, we document dorsal fin disfigurements, body deformities and confirm the identity of a whale mortality. Our study reveals the need for the long-term monitoring of Rice’s whale individuals, especially presumed mothers and calves, and to further investigate potential human threats to this population using photo-identification techniques.

## Introduction

The Rice’s whale (*Balaenoptera ricei*) is the only species of baleen whale known to reside year-round in the Gulf of America (formerly U.S. Gulf of Mexico, hereafter referred to as the Gulf) [1,2]. Rice’s whales are one of the most endangered species of cetaceans in the world and are listed as Endangered under the United States Endangered Species Act [2] and as Critically Endangered on the IUCN Red List [3]. Factors that contribute to their endangered status include a small population size (N = 51 [20–130, 95% Confidence Interval] [4], extremely low genetic diversity [1], and vulnerability to multiple anthropogenic threats [5].

To date, Rice’s whales have been primarily found in the Gulf between 100 m and 400 m water depths [6] but a few confirmed strandings along the U.S. Atlantic coast have been reported [2]. These whales are most commonly observed in the northeastern Gulf, off the west coast of Florida, in an area referred to as the Rice’s Whale Core Distribution Area [7] ([hereafter referred to as the Core Area – [6]]. However, modeling studies in the Gulf have identified suitable habitat throughout the continental shelf break of both U.S. and Mexican waters [6]. In the western Gulf, Rice’s whales have been detected visually and acoustically [8,9], while, in Mexican waters, Rice’s whales have been detected only acoustically.

We lack precise population size estimates, knowledge of life history, reproductive parameters, and the extent to which Rice’s whales move within and out of the Core Area. Due to the heavy industrialization of the Gulf, Rice’s whales are threatened by vessel strikes, noise from energy exploration and other extractive activities, oil spills and associated response actions, ingestion of marine debris, and fisheries interactions, as well as the effect of environmental variability at short and long-time scales on their prey [2,5,10].

Photo-identification (photo-ID) techniques can be used to estimate population abundance using capture-recapture methods [11,12], describe home ranges [13], characterize movement patterns [14], quantify individual associations [15], and evaluate individual and population health [16]. The types of features used for photo-ID vary by species but generally, dorsal fin attributes, such as scars, nicks, and notches are used as the primary features for multiple delphinid [17] and baleen whale species: fin whales (*Balaenoptera physalus*) [18]; Bryde’s whales (*Balaenoptera brydei*) [19]; minke whales (*Balaenoptera acutorostrata*) [14]. For fin and blue whales (*Balaenoptera musculus*), the shape of the dorsal fin alone is distinctive enough among individuals that it can be used as a primary identifying attribute [20,21]. On the body of rorqual whales, pigmentation patterns such as the chevron in fin whales [20] and thoracic patches on minke whales [22], as well as scars can be used as secondary identification features that assist matching [14,23,24]. Collectively, dorsal fin attributes and body marks increase the accuracy and number of matches within a photo-ID catalog [14,20,21]. Multiple factors influence the ability to match individuals, including photographic quality [25], individual distinctiveness [12], mark persistence [26], examiner confidence [27] and experience [28].

The source of dorsal fin attributes and body marks used in photo-ID studies can have natural and/or anthropogenic origins. Natural marks usually originate from interactions with conspecifics and predators [24]. One of the most commonly used natural body marks are depressed oval-shaped wounds or scars that are attributed to predation by cookiecutter sharks (*Isistius* spp.) [29], which are known to predate on multiple species of cetaceans worldwide [30–32], including in the Gulf [33]. On the other hand, linear mutilations and clean-edge cuts, especially involving the apex and the base of the dorsal fin and the peduncle of cetaceans, are suspected to have an anthropogenic origin, such as interactions with fishing gear and vessel collisions [34–38].

Rice’s whale photographs, associated sighting information and biopsy samples, have been collected during vessel-based research surveys conducted by the U.S. National Marine Fisheries Service’s Southeast Fisheries Science Center (SEFSC) in the Gulf since the early 2000s [6,39]. Most studies were large vessel-based line-transect surveys with the primary goal of collecting sighting data to determine the distribution and abundance of oceanic cetaceans in U.S. waters [6]. Additional surveys dedicated to studying Rice’s whales in the Core Area were also conducted and included efforts to photograph, biopsy sample, and tag whales [40,41].

The primary goal of this study was to develop a photo-ID catalog for Rice’s whales incorporating associated genetic data from samples (genotyping and sex) ultimately creating sighting histories for the identified whales. Secondary goals were to characterize group composition with a focus on presumed mother and calf pairs and to characterize the dorsal fin attributes and body marks used for individual identification and matching.

## Materials and methods

### Data collection

We collected data on survey effort, baleen whale sightings, and biopsy samples during 16 vessel-based surveys conducted by the SEFSC in the Gulf between 2004 and 2019. Five surveys between 2010 and 2019 (i.e., GU1005, GU1505, GU1802, GU1806, and GU1901) were targeted studies of Rice’s whales, and effort was concentrated in the Core Area. For four surveys (GU0603, GU1102, GU1202, GU1605), only the portion that transited through the Core Area was considered. Effort inside the Core Area was calculated by clipping the total km of surveyed trackline for each survey with the shapefile for the Core Area [7] using ArcGIS 10.5 (World Geodetic System 1984 - WGS84/ Universal Transverse Mercator – UTM – zone 16N). Survey effort included kilometers covered during active searching for cetaceans.

During the surveys, two or four trained marine mammal observers scanned the water using 25x (“big-eyes”) binoculars in search of whales; the number of observers depended on the goal of the survey [e.g., 39,41]. A sighting consisted of one or more whales in the same general area and at the same time. Sightings were designated as Rice’s whale if the characteristic three ridges on the rostrum of at least one animal were observed. Other sightings were recorded at higher taxonomic levels and included unidentified baleen whale (*Balaenoptera* spp.), sei or Rice’s or fin whale (*Balaenoptera borealis/ricei/physalus*) if none of the three species could be ruled out, or sei or Rice’s whale (*Balaenoptera borealis/ricei*) if fin whales were ruled out. Observers estimated group size and noted composition for each sighting. If multiple group sizes were estimated for the same sighting, the average was used. For composition, the terms “adult” and “calf” were used to generally describe whales that appeared larger or smaller in relation to one another; and are not confirmation of sexual maturity, actual body length measurements, or other age-class determinations.

Photographs of whales were taken from the flying bridge or bow of the ship and, when feasible, from a 7-m rigid-hulled inflatable boat (RHIB) deployed from the ship. All photos were taken using high-resolution, digital single-lens reflex (DSLR) cameras equipped with a 100–400 mm or 70–300 mm zoom telephoto lens. Photographs taken from the RHIB may have been concurrent with biopsying and/or tagging efforts.

In addition, this study included three opportunistically collected photos of whales in the Gulf provided by members of the public, researchers from Oregon State University (O.S.U.) Marine Mammal Institute, and stranding responders from the Florida Fish and Wildlife Conservation Commission’s Marine Mammal Pathobiology Laboratory (FWC-MMPL).

The Southeast Fisheries Science Center was authorized to conduct marine mammal research activities under the National Marine Fisheries Service (NMFS), Marine Mammal Protection Act (MMPA), and Endangered Species Act (ESA) permit numbers 779−1633 (2002–2014), 14450 (2014–2019), and 21938 (2019–2024). In addition, this study was carried out in strict accordance with the NMFS Animal Use and Care Policy and associated Institutional Animal Care and Use Committee (IACUC) guidelines, Cetacean Stock Assessment Research in the Northwest North Atlantic, Gulf of Mexico, and Caribbean Sea (protocol numbers: Atlantic-2013–001, ATL-2017–001, and ATL-2020–002). All Rice’s whales were biopsied in U.S. waters. Biopsy sampling involved collecting small plugs of skin and blubber using modified crossbows, a method that fell within Animal Welfare Category C (little or momentary pain or discomfort, no use of pain-relieving drugs) and approved by the NMFS Atlantic IACUC. Throughout the biopsy sampling efforts, animals were monitored for behavioral changes and potential reactions to the biopsying procedure.

### Photo selection and processing

Photographs were collected in .JPG file format and stored according to unique sighting numbers within each survey’s dataset. Two examiners, KPB and LAD, both with previous photo-ID experience in the field and laboratory, selected and evaluated the photos. A triage of images was done by selecting photos that best showed different portions of the body of the whales, from multiple sides and angles, if available. If it was possible to see dorsal fin attributes and/or marks on the body of the whales, even if distant, the photos were selected. Subsequently, sequential photos of whale surfacings were separated into subfolders with temporary identifiers (e.g., individual A). Once all photos of a sighting were processed as such, we looked for whales that may have been photographed multiple times during the same sighting (i.e., during different surfacings) and combined the photographs under the single temporary identifier. For this initial identification of individuals, in addition to dorsal fin attributes and body marks, we also used features typically considered less “stable” (long-term) such as the presence of the barnacle *Xenobalanus* in the dorsal fin and areas of different coloration in the skin of the whales to assign matches within the same day. Some of the images were cropped and enhanced, and the photographs that best showed the dorsal fin and any other markings on the body of the whale (both sides and multiple angles, if available) were imported into FinBase. FinBase is a cataloging program, created for bottlenose dolphin (*Tursiops truncatus*) photo-ID analysis that uses characteristics of the dorsal fin as primary attributes to classify and sort individuals [42]. Whales were classified as having distinct, marginally distinct, or non-distinct dorsal fins. Whales with distinct dorsal fins presented conspicuous attributes evident even in poor-quality photos; whales with marginally distinct fins showed inconspicuous attributes; and whales with non-distinctive fins showed no attributes that alone would allow for identification and matching [43]. In addition to dorsal fin attributes, a limited number of features based on body marks were also available in FinBase (e.g., “Peduncle Scar/Notch”, or “Anterior to Fin”, “Skin lesion”) [43]. To record information on additional marks, a searchable spreadsheet was created [similar to 44] indicating the view (e.g., left, right, caudal, etc.), type of mark (e.g., cookiecutter shark bite scar, injury, nodules, etc.), a more specific location on the body (e.g., dorsum, below dorsal fin, peduncle, etc.) and the number of cookiecutter shark bite scars seen.

All photographs were evaluated based on focus (excellent-1, moderate-4, or poor-9) and contrast (ideal-1, reasonable-3, or poor-9) [S1 adapted from 2,44,45]. An overall photo quality (PQ) was obtained by the sum of both parameters that classified each image as excellent (2–4), good (5–7), fair (10), or poor (>11). In addition, images that contained dorsal fins, either alone (cropped image that showed dorsal fin only) or that included other body parts were also evaluated based on dorsal fin angle in relation to the camera (perpendicular-1, slight-2, or oblique-9) and leading and trailing edges visibility in the frame (fully visible-1 or partially obstructed-8); scores for angle and visibility were added to the overall score based on focus and contrast and also classified images as excellent (4–6), good (7–9), fair (10–11), or poor (12–35) [S1 adapted from 11,44,45].

A match involved identifying an individual whale in photographs originating from different sightings, recorded in the same or different surveys. For whales matched in multiple sightings (re-sightings), timespan was the difference in days between the first and last sightings, irrespective of the number of times an individual was matched. To confirm matches, the two-person approach for initial matching and verification was conducted per standard photo-ID study protocols [43]. Furthermore, for this study, matches were confirmed using the following criteria: 1- after matching and verification cases were individually discussed and both examiners must agree on a match; 2- one feature must be conspicuous (e.g., deep notch on the dorsal fin or a substantial body mark); and 3- multiple body marks must be used when matching whales with non-distinctive dorsal fins.

### Genotyping and sex determination

Thirty three Rice’s whale biopsy samples (skin and blubber) were collected from the RHIB whenever survey conditions and weather allowed [S3 File; for biopsy methods see 46]. A skin sample and dorsal fin photos were also obtained from the necropsy examination of a whale that stranded in the Florida Everglades in 2019 [2]. Genetic sequencing of the mitochondrial DNA control region was performed on all samples to verify species; sexing was also performed on all skin samples [1,2], except sexing on the sample from the stranded whale, which was determined by physical examination (Boyd, personal communication). In addition, all samples were previously genotyped at 17 microsatellite loci [1,2] to identify samples collected from the same animal. Microsatellite Toolkit [47] was used to search for individuals with identical multilocus genotypes and estimated the probability of identity P(ID) and the more conservative P(ID)sib [48] using GenAlEx v6.5 [49]. Samples identified as having identical genotypes were also checked for conserved sex and control region sequence.

After the photo matching process was completed by both examiners, results from genotyping were compared with the photographic matches to verify that no different genotypes were associated with the same photographically identified individual. In addition, sex of the individuals was incorporated in the catalog.

## Results

**Fig 1:**
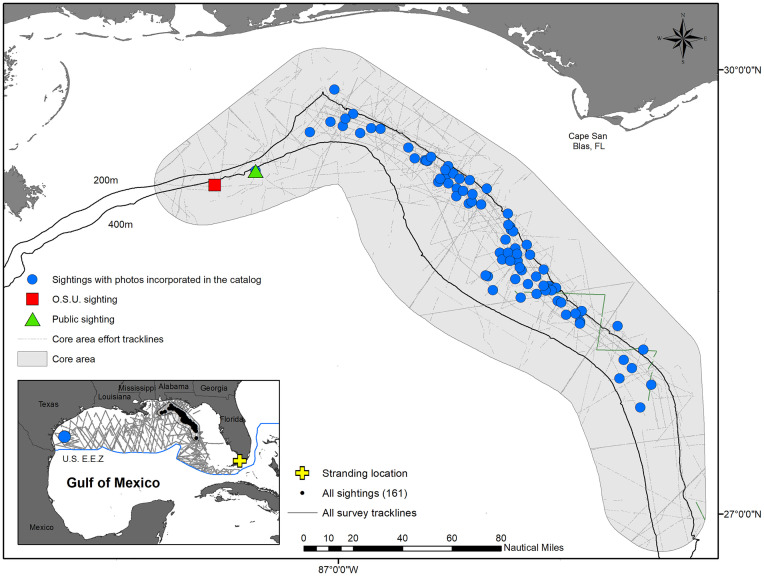
Rice’s whale sightings with photos incorporated into the catalog and survey tracklines performed in the Core Area during the 16 SEFSC surveys. Inner map: total survey tracklines and Rice’s whale sightings, including one off the coast of TX seen in 2017 and the stranding location off FL in 2019.

Between 2004 and 2019, we covered nearly 17,000 km of trackline (N = 16 surveys) inside the Rice’s whale Core Area and recorded 161 baleen whale sightings. Group size ranged from one to 11 whales (mean = 2.6, SE = 2.1), with 43% of sightings consisting of a single animal. Of the total sightings, 107 sightings were visually confirmed to be Rice’s whales and included 80 sightings with photographs incorporated into the catalog; for the remaining 27 sightings, photos were too far away and/or did not show enough of the body of the whale(s) to be useful for photo-ID (Table 1 and Fig 1).

**Table 1:** Survey effort in km and number of whale sightings during the 16 research surveys conducted by the SEFSC between 2004 and 2019. Bold designates surveys dedicated to studying Rice’s whales with most or all effort in the Core Area.

Year	Season	Survey	Core Area Effort (km)	Total survey Effort (km)	Core Area to total survey ratio	Num. baleen whale sights.	Num. Rice’s whale sights.	Num. sights. with photos	Num. sights. in catalog
2004	Spring	GU0402	876	6,927	13%	5	4	3	3
2006	Summer	GU0603*	86	193	45%	1	1	0	0
2007	Summer	GU0704	1,350	4,917	27%	3	3	2	3
2009	Summer	GU0903	389	4,578	9%	3	3	3	2
2010	Summer	GU1003	652	2,681	24%	2	2	2	1
**2010**	**Fall**	**GU1005**	**1,923**	**2,267**	**85%**	**5**	**5**	**5**	**5**
2011	Summer	GU1102*	178	359	49%	4	4	4	4
2012	Summer	GU1202*	694	4,153	17%	2	2	2	0
2015	Summer/Fall	GU1505	2,481	3,563	70%	22	21	17	11
2016	Summer	GU1605*	199	332	60%	3	3	3	2
2017	Summer	GU1703	942	7,289	13%	3	3	3	2
2018	Winter	GU1801	963	5,814	17%	2	1	2	1
2018	Summer	GU1802	796	796	100%	26	12	14	11
2018	Summer/Fall	PC1805	703	6,473	11%	2	0	1	0
2018	Fall	GU1806	960	960	100%	18	4	6	4
2019	Summer	GU1901	3,572	3,624	99%	60	39	40	31

* surveys that transited through the Rice’s whale Core Area.

Based on focus and contrast scores combined (i.e., overall PQ), 82% of all photos included in the catalog were of excellent (41%) or good (41%) PQ; fair and poor photographs were 7% and 11%, respectively. For photographs that contained the dorsal fin, when adding the scores for angle and visibility, 27% of the photos were of excellent PQ, 31% good, 4% fair and 38% poor.

### Individually identified whales

**Fig 2:**
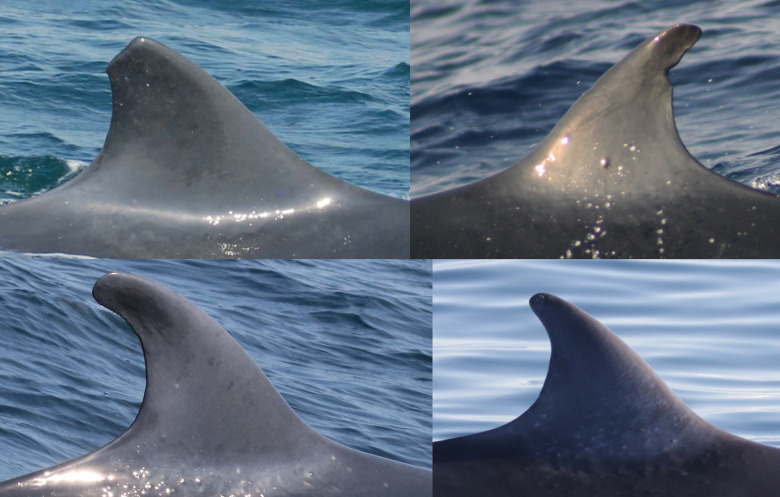
Dorsal fin attributes used for individually identifying and matching Rice’s whales. Catalog ID 1000 “Stumpy” with a distinctive dorsal fin caused by a laceration (top left), catalog ID 6000 “Jean Jacques” with a distinctive dorsal fin caused by a deep round notch on the upper trailing edge (top right), catalog ID 12005 “Squiggles” with a marginally distinctive dorsal fin with small and shallow nicks on the upper trailing edge (bottom left), and catalog ID 12003 “Edna “ with a marginally distinctive dorsal fin with a unique overall shape (bottom right).

**Fig 3:**
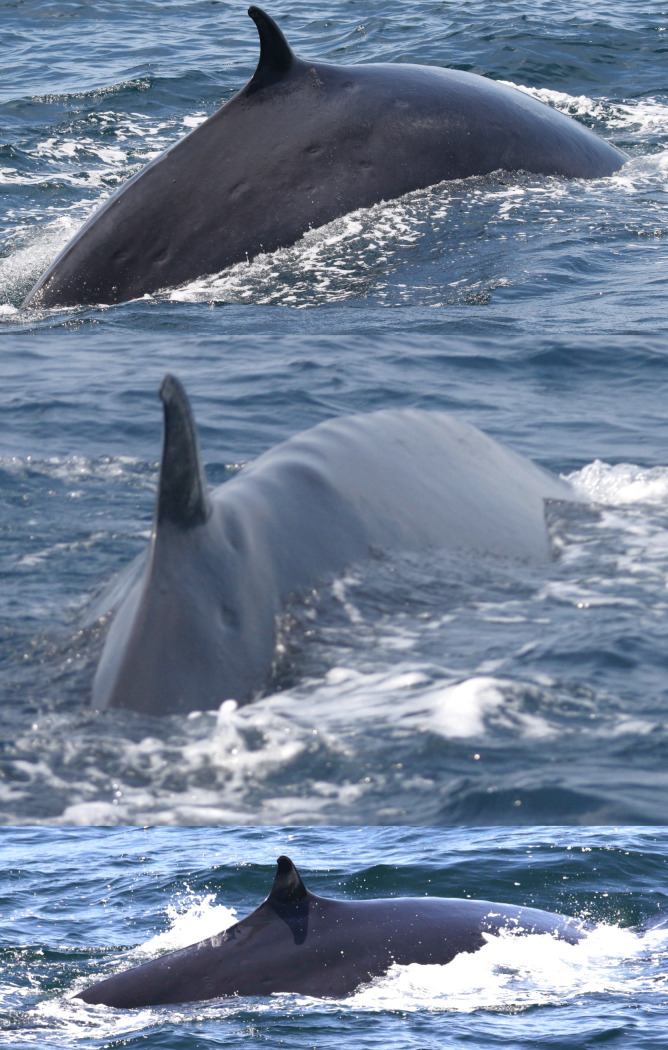
Whales with non-distinctive dorsal fins primarily matched by cookiecutter shark bite scars on their bodies. Catalog ID 20031 “Splash” with multiple bite scars on the body (top), catalog ID 20001 “Scyther” with a few bite scars under the dorsal fin and a vertebral anatomical malformation (center), and catalog ID 20023 “Jo Jo” with multiple bite scars behind the dorsal fin (bottom).

**Fig 4:**
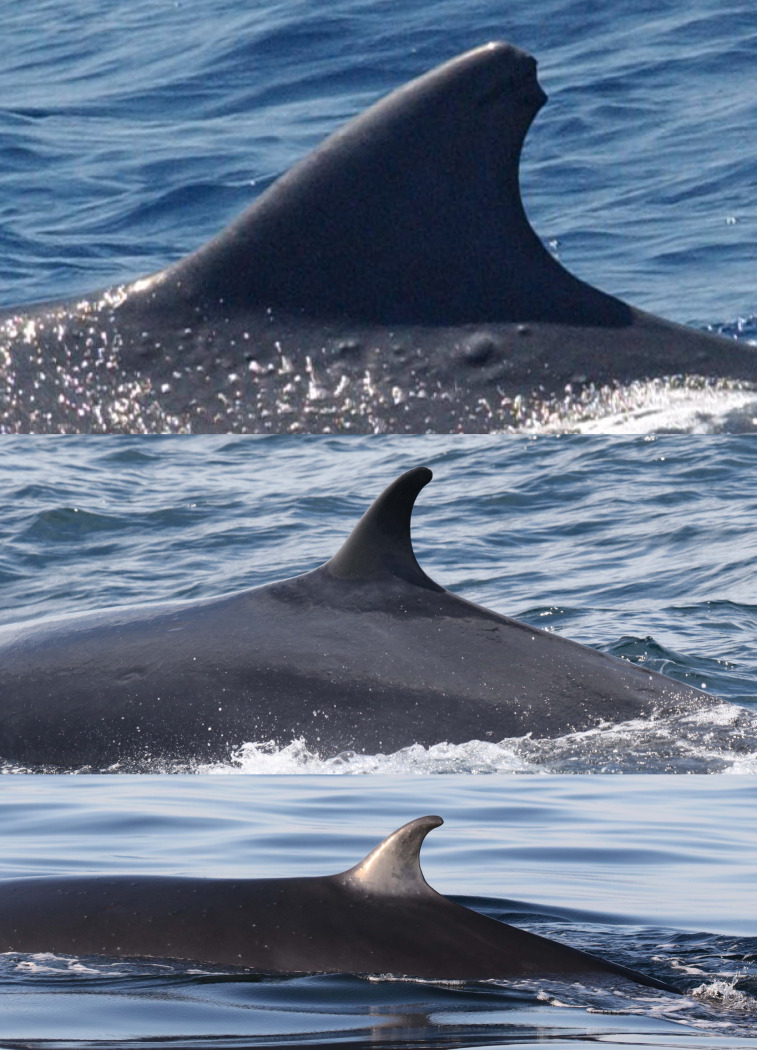
Secondary features used for identifying and matching Rice’s whales. Catalog ID 1000 “Beaker” displaying nodules of varying sizes (top), catalog ID 20031 “Splash” with a gap on the peduncle (center), and catalog ID 20014 “Milky Way” with white nodules and nicks on the peduncle (bottom).

A total of 31 Rice’s whales were individually identified based on dorsal fins attributes and/or body marks. Eleven whales were categorized as having distinctive dorsal fins, 12 as marginally distinctive fins and eight had non-distinctive fins (Table 2). For whales with distinctive and marginally distinctive dorsal fins, lacerations, nicks and notches and the overall shape of dorsal fins were the primary features used for identification and matching (Fig 2). Whales with non-distinctive dorsal fins displayed no attributes on the leading or trailing edges of the fin. Therefore, the primary feature used for identifying and matching were cookiecutter shark bite scars on the body (Fig 3). In addition, deformities, skin nodules, nicks and gaps on the peduncle were secondary features used to assist in matching multiple whales (Figs 3 and 4).

**Table 2:** The 31 individually identified Rice’s whales, their fin type (Dist. = distinctive, Marg. = marginally distinctive and ND = non-distinctive), sex (if known), number of sightings matched, time span in days between first and last sightings; along with primary and secondary features used for matching (Anat. malf. = anatomical malformation, Cc = cookiecutter shark bite scar, DF lac. = dorsal fin laceration, DF NN = dorsal fin nicks and notches, DF pigm. = dorsal fin pigmentation, Nod. = nodules, ped. nicks = peduncle nicks and gaps). Bold indicates genetic duplicates.

Catalog ID	Alias	Fin Type	Sex	Num sights.	1st sight.	Last sight.	Time Span (days)	Prim. feat.	Sec. feat(s)
1000	Beaker	Dist.	F	3	5-May-04	30-Jul-19	5564	DF lac.	Cc scar, nod.
1001	Stumpy	Dist.	M	5	30-Jun-07	30-Jul-19	4413	DF lac.	Ped. nicks
2000	Chip Tip	Dist.	F	10	17-Jun-10	21-Jul-19	3321	DF lac.	Cc scar
2001	Scar	Marg.	U	5	22-Jun-19	30-Jul-19	38	DF NN	DF pigm.
2002	Cliff	Marg.	U	1	3-Jun-19	–	0	DF shape	Cc scar, nod., ped. nicks
6000	Jean Jacques	Dist.	M	2	30-Jun-07	31-Jul-11	1492	DF NN	Cc scar, nod.
6001	El Capitan	Dist.	U	8	31-Jul-11	23-Jun-19	2884	DF NN	Cc
6002	Patty	Marg.	F	5	4-Sep-15	15-Jul-19	1410	DF NN	Scar, Cc scar
6003	Joker	Dist.	U	4	23-Jul-17	26-Jun-19	703	DF lac.	Cc
6004	Scoop	Marg.	F	9	18-Oct-10	30-Jul-19	3207	DF NN	Cc
7000	Buddy	Dist.	U	6	16-Oct-10	29-Jul-19	3208	DF NN	Cc scar, nod.
7001	Gonzo	Dist.	F	8	1-Jul-07 *	30-Jul-19	4412	DF NN	Scar, Cc scar
7002	Mystery	Dist.	U	2	4-Sep-15	24-Sep-15	20	DF NN	None
7003	Witch Hazel	Dist.	M	2	19-Nov-18	28-Jan-19 **	70	DF NN	Cc
8000	Achilles	Dist.	U	3	20-May-04	23-Jun-19	5512	DF NN	Scar, Cc scar
9000	Lucky	Marg.	U	2	24-Jul-19	31-Jul-19	7	Injury	Cc
12003	Edna	Marg.	U	7	25-Jun-18	23-Jul-19	393	DF shape	Scar, Cc scar
12004	Uncle	Marg.	U	3	20-Sep-15	29-Jul-19	1408	DF NN	Cc
12005	Squiggles	Marg.	U	1	30-Jun-18	–	0	DF NN	Cc
12006	Galactica	Marg.	U	1	21-Jun-19	–	0	DF NN	Cc scar, nod.
12008	Dig Deeper	Marg.	M	3	1-Aug-11	30-Jul-19	2920	DF shape	Cc
12009	Ms. Clean	Marg.	F	4	4-Sep-15	31-Jul-19	1426	DF shape	Cc
20001	Scyther	ND	F	2	30-Jun-07	24-Jul-11 ***	1485	Anat. malf.	Cc
20004	Dot	ND	M	4	1-Jul-09	25-Jul-19	3676	CC	Nod.
20010	Trouble	ND	M	6	31-Jul-11	19-Nov-18	2668	CC	Nod.
20014	Milky Way	ND	U	2	20-Sep-15	30-Jul-19	1409	Nod.	Ped. nicks
20023	Jo Jo	ND	U	2	23-Jul-17	23-Jun-19	700	CC	Cc scar, ped. nicks
20026	Bump	ND	U	3	15-Mar-18	5-Jun-19	447	Nod.	Cc scar, ped. nicks
20031	Splash	ND	F	2	4-Sep-15	3-Jul-18	1033	CC	Cc scar, ped. nicks
20035	Spider Bite	ND	M	4	15-Sep-15	31-Jul-19	1415	CC	Cc scar, nod., ped. nicks
20047	Barry	Marg.	F	2	20-Sep-09 ****	22-Jun-19	3562	DF shape	Cc

*no photographs; **stranding; ***O.S.U. survey; ****public sighting.

Of the 31 individually identified whales, 28 were matched multiple times (re-sighted) between two and 10 sightings and with a timespan of seven days to more than 15 years (5500+ days) (Table 2 and S2 File).

**Fig 5:**
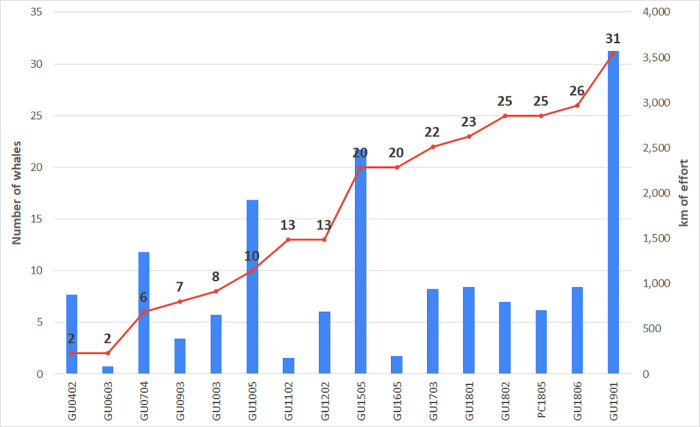
Discovery curve of the cumulative number of individually identified Rice’s whales (red) by the km of effort (blue) surveyed inside the Core Area during all SEFSC surveys between 2004 and 2019.

Across all SEFSC surveys, the cumulative number of individually identified whales continued to grow, with a somewhat gradual increase up to 13 whales until the survey in 2015 (GU1505), when seven new individuals were photographed. Between 2015 and 2018 (GU1806), roughly one new individual was photographed per survey until the survey in 2019 (GU1901) when five new whales were identified (Fig 5).

### Genetically identified individuals

Of the 31 photo-identified whales, 16 were biopsied and included nine females and seven males (Table 2). An additional nine whales were biopsied, including five females and four males (S2 File) that were not able to be matched (individually identified) through photographs due to the lack of conspicuous features in either the dorsal fin and/or body. All 25 sampled whales were considered unique individuals based on genomic data (S3 File).

**Fig 6:**
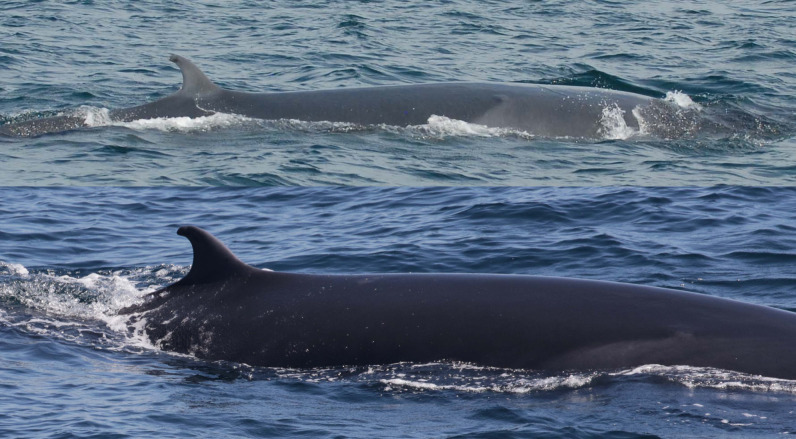
Catalog ID 12008 “Dig Deeper” a whale missed during the initial photographic matching and matched by genetic samples collected in 2011 (top) and 2019 (bottom). Note the slight indentation on the dorsal fin (bottom) and possible cookiecutter shark bite scar partially merged with the chevron on both images.

Six of the 16 photo-identified whales were determined to have been biopsy sampled multiple times across surveys because of matching genotypes and all except one were successfully identified photographically during the initial matching process (Table 2 and S3 File). The duplicate biopsy sample that was genetically matched but was not photographically matched in photographs taken during biopsy efforts in 2011 and 2019 was Catalog ID 12008 “Dig Deeper”, a male. Based on the genetic match results, we re-evaluated the photos and identified a slight indentation on the dorsal fin and a possible cookiecutter shark bite scar that allowed us to match this individual’s photo from 2011 to photos taken during 2019 sightings (Fig 6 and S2 File). The other whales with multiple genetic samples displayed unaltered dorsal fin attributes and/or body marks between sampling events when photographs were available.

**Fig 7:**
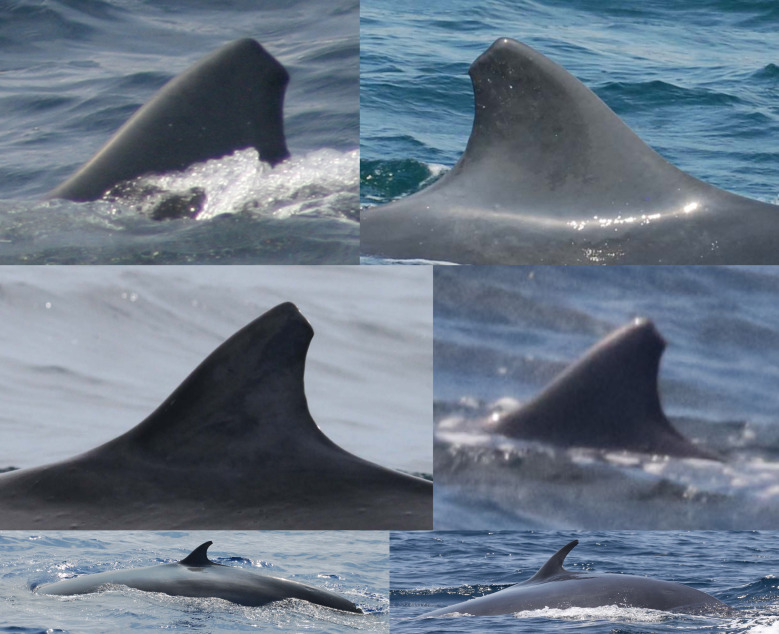
Catalog ID 1001 “Stumpy” showing the unchanged dorsal fin photographed in 2007 (top left), 2011 (top right), 2015 (center left) and 2019 (center right). Catalog ID 2000 “Chip Tip” photographed in 2010 (bottom left) and 2019 (bottom right), showing the unaltered dorsal fin profile and the cookiecutter shark bite scar on the left peduncle on both images.

Catalog ID 1001 “Stumpy”, a male, was re-sighted five times between 2007 and 2019 with an unchanged dorsal fin; this individual was biopsied in 2011 and 2015 (Fig 7 and S2 File). For catalog ID 2000 “Chip Tip”, a female with the highest number of re-sightings (10), the dorsal fin remained unchanged, in addition, it was possible to see the same cookiecutter shark bite scar on the peduncle during the two biopsy efforts in 2010 and 2019 (Fig 7 and S2 File).

**Fig 8:**
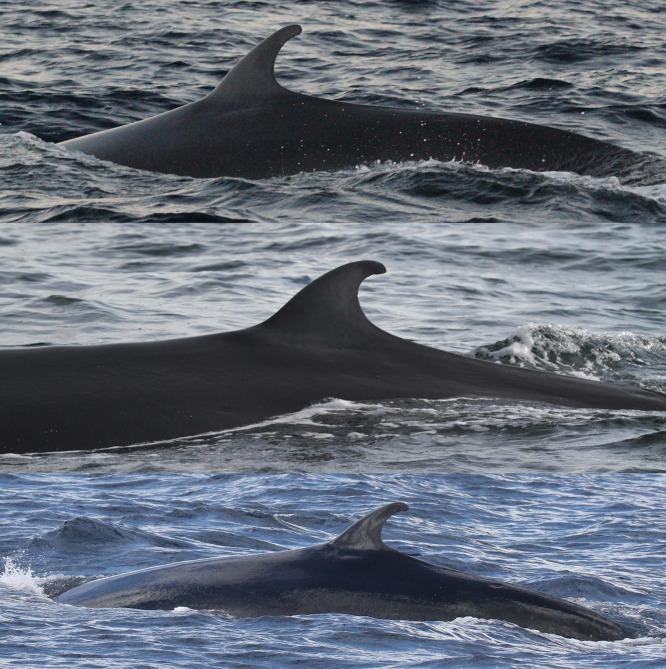
Catalog ID 6004 “Scoop” photographed in 2010 (top), 2017 (center) and 2019 (bottom). The cookiecutter shark bite scar on the left peduncle is seen in all images; the shallow notch on the upper trailing edge of the dorsal fin is seen only in 2017 and 2019.

Catalog ID 6005 “Scoop”, a female, was first photographed and biopsy sampled in 2010. In the 2010 photos it was possible to see a cookiecutter shark bite scar on the peduncle but the dorsal fin did not present the shallow notch subsequently photographed in 2017 and 2019, concurrent with biopsy samples; the cookiecutter shark bite scar on the peduncle remained unchanged (Fig 8 and S2 File).

**Fig 9:**
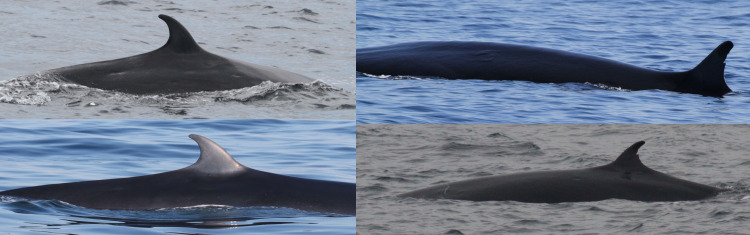
Catalog ID 12009 “Ms. Clean” displaying the unchanged dorsal fin and multiple cookiecutter shark bite scars on the body photographed in 2015 (top left) and 2019 (bottom left). Catalog ID 7001 “Gonzo” displaying the unchanged highly distinctive dorsal fin, a cookiecutter shark bite scar and a large round scar on the dorsum in 2010 (top right) and 2011 (bottom right).

For catalog ID 12009 “Ms. Clean” biopsy samples were collected only one day apart in 2019. In previous photographs taken in 2015, it was possible to see the unchanged dorsal fin profile and multiple cookiecutter shark bite scars that remained stable over time (Fig 9). For the final genetic match, catalog ID 7001 “Gonzo”, photos from the first biopsying event in 2007 were not available but subsequent photos taken in 2010, 2011, 2018, and 2019 showed the unchanged highly distinctive dorsal fin, multiple cookiecutter shark bite scars and a scar on the left dorsum of the whale that remained unchanged over time (Fig 9 and S2 File).

### Potential mother and calf pairs

For sightings that included individually identified whales of known sex, the group composition varied from single males and single females to combinations of both and unknown sexes. Of the 161 sightings, only 10 included notes and photos indicating the presence of two associated whales with one being larger than the other and therefore, potential mother and calf pairs.

**Fig 10:**
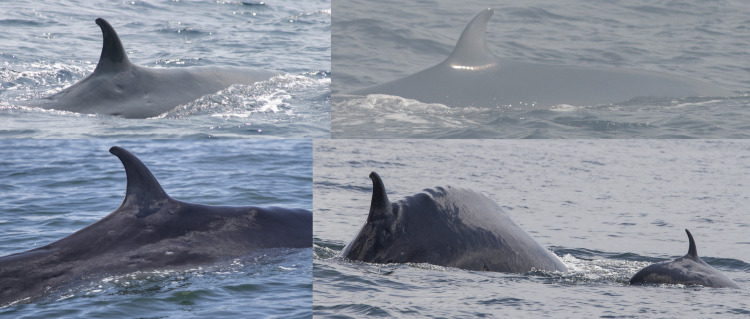
Catalog ID 20001 “Scyther”, a female, individually identified by the multiple cookiecutter shark bite scars and a vertebral anatomical malformation, photographed in 2007 (top left) and 2011 (bottom left). The presumed calf from 2007, catalog ID 20002 (top right) is shown with a non-distinctive dorsal fin; the bottom right image shows “Scyther” with the presumed calf, catalog ID 20049, photographed in 2011 by O.S.U.

During one sighting in 2007, a presumed mother and calf pair was photographed during biopsy efforts (S2 File). Both whales were biopsy sampled. The adult, a female, was identified by cookiecutter shark bite scars and an anatomical malformation on the vertebrae (Fig 3) as catalog ID 20001 “Scyther”. The smaller individual was an unidentified male (catalog ID 20002). Preliminary genetic analysis of the samples collected from these two whales indicated that due to the inherent low genetic diversity of this population, it was not possible to accurately determine the extent to which they were related using microsatellite data (Wilcox Talbot, personal communication). In 2011, “Scyther” was re-sighted accompanied by a small whale (catalog ID 20049) in photographs provided to us by researches from O.S.U. (Fig 10). 

**Fig 11:**
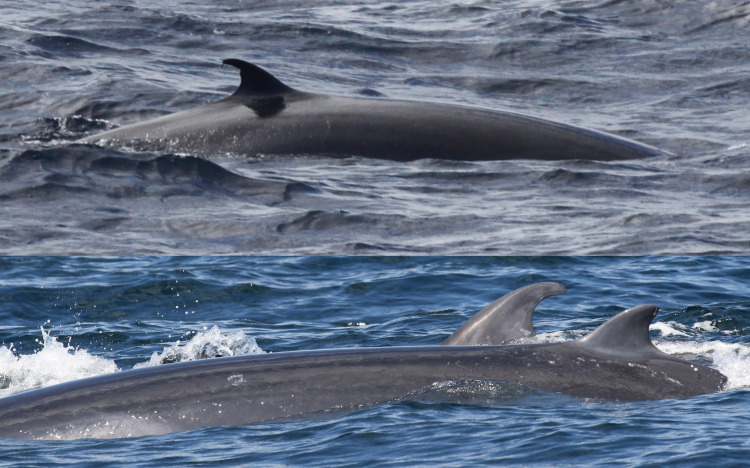
Catalog ID 7002 “Mystery”, presumed calf of catalog ID 12009 “Ms. Clean”, photographed in 2015 (top). Catalog ID 6004 “Scoop” (bottom and back) and presumed calf catalog ID 2001 “Scar” (bottom and front) photographed together in 2019; note the small notch and depigmentation on the apex of “Scar’s” dorsal fin.

In 2015 a pair of whales was photographed during two sightings 20 days apart (S2 File). Notes indicated that one whale was approximately, “⅔ the size of the adult”. Throughout the photographic effort, it became apparent that the pair was strongly bonded, as they swam side-by-side in echelon position and eventually away together from the area (Aichinger Dias and Barry, personal observation). The adult was identified as catalog ID 12009 “Ms. Clean”, a female (Fig 9). The smaller whale was cataloged as catalog ID 7002 “Mystery” and despite having a deep notch on the dorsal fin has not been re-sighted (Fig 11); whereas, “Ms. Clean” was re-sighted alone in 2019, nearly four years later (S2 File).

**Fig 12:**
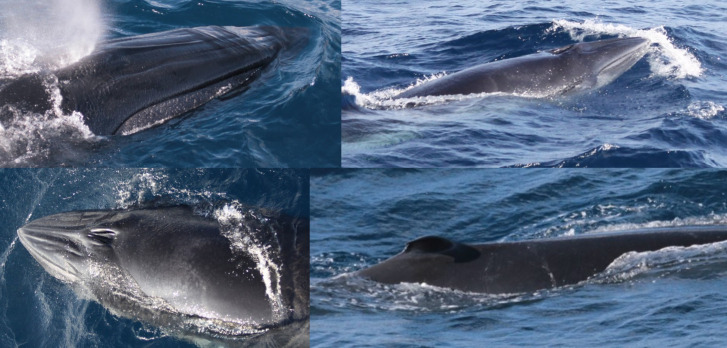
Catalog ID 6004 “Scoop” (adult female) with a relatively large and smooth rostrum (top left) and her presumed calf, catalog ID 20021 with a relatively small rostrum (top left). Details for catalog ID 20021: mottling on the skin and roughened rostrum (bottom left), and bent-over dorsal fin (bottom right).

In a 2016 sighting, notes indicated the presence of a pair of whales, “one being a little over ½ the length of the adult”. The adult was identified as catalog ID 6004 “Scoop”, a female originally observed in 2010 alone (Fig 8). The presumed calf, catalog ID 20021, displayed mottling of the skin and a bent over dorsal fin (Fig 12). Further examination of the photographs indicated potential differences in the rostrum of the two whales, with the adult showing a smoother and relatively longer rostrum than the presumed calf (Mullin, personal communication; Fig 12).

In 2017, catalog ID 6004 “Scoop” was re-sighted alone. In 2019, “Scoop” was seen multiple times within a 1.5-month period with a smaller individual (S2 File). The smaller whale displayed a marginally distinctive dorsal fin with a small notch and some pigmentation on the apex and was cataloged as catalog ID 2001 “Scar”(Fig 11).

### Dorsal fin disfigurements, body deformities, and mortality

Many of the dorsal fin attributes that allowed us to identify individual Rice’s whales were linear and/or clean-edge cuts with loss of tissue (laceration) that considerably changed the profile of the fin. Four whales presented linear lacerations, ranging from complete removal of the tip to alterations of the apex, as displayed by catalog IDs 1001 “Stumpy” (Figs 2 and 7) and 2000 “Chip Tip” (Fig 7). In addition, six whales presented healed deep triangular or round clean-edge cuts (notches) on the trailing edge of their fins as seen in catalog IDs 6000 “Jean Jacques” (Fig 2), 7001 “Gonzo” (Fig 9), and 7002 “Mystery” (Fig 11).

**Fig 13:**
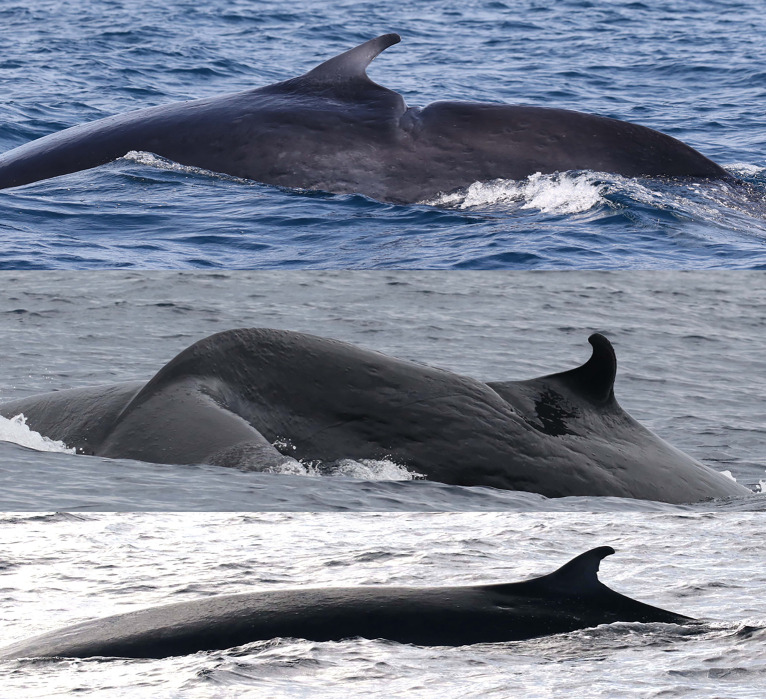
Catalog ID 9000 “Lucky” photographed in 2019 with a large healed scar on the peduncle indicative of a vessel strike (top and center). Catalog ID 7003 “Witch Hazel” photographed alive in 2018 displaying the distinctive dorsal fin and sunken dorsum, indicative of emaciation (bottom).

Two whales had structural deformities to their bodies. Catalog ID 20001 “Scyther” was a female seen in 2007 and 2011 with a dislocated vertebra, cranial to the dorsal fin, causing an asymmetric anatomical malformation along the dorsum (Figs 3 and 10). Catalog ID 9000 “Lucky” was photographed twice in 2019 with a large healed transverse scar across the peduncle (Fig 13 and S2 File).

The whale that stranded in the Florida Everglades in 2019 was identified by its distinctive dorsal fin as catalog ID 7003 “Witch Hazel”, a whale first photographed in 2018 (Fig 13 and S2 File). Necropsy examination determined this whale to be a 11.26 m, subadult (presumptive), male with a thin body condition and multiple fresh and healed wounds indicative of cookiecutter shark bites. In addition, a sharp piece of hard plastic measuring 6.6 x 6.2 x 0.2 cm was found in the stomach surrounded by mild hemorrhage and was considered a potential contributing factor in the animal’s death in the absence of evidence of a severe disease process or major traumatic injury [Boyd, personal communication; 2].

## Discussion

Similar to other baleen whale species, attributes like nicks, notches, and lacerations were present in Rice’s whale dorsal fins and could be used as primary features for identifying and matching individual whales over time [14,19,20,26,50]. Deep notches and lacerations were the main attributes in making Rice’s whale dorsal fins highly distinguishable. Unlike fin and blue whales, which show strongly contrasting pigmentation patterns and distinguishable dorsal fins based on overall shape alone [20,21], we found that Rice’s whale body pigmentation, such as chevrons, were not readily apparent or bold like fin whales. Likewise, the overall shape of dorsal fins varied little among individual Rice’s whales. We identified five Rice’s whales for which the overall shape of the dorsal fin varied enough to result in a unique profile; still, the variation was not as apparent as that of fin or blue whales. We believe that the lack of both conspicuous body marks and a strong dorsal fin profile, having only a slight indentation on the apex of the fin, resulted in catalog ID 12008 “Dig Deeper” being missed during the initial photographic matching. This whale was matched genetically and after re-evaluating the photos, a likely cookiecutter shark bite scar, partially merged with pigmentation of the chevron, allowed us to match this individual in another sighting in 2019 (S2 File). This case is a good example of how genotyping, when available, can supplement the photo matching.

Combining photo-ID and genotyping results can be used to more precisely estimate population abundance during capture-recapture studies. Comparing estimates from genetic and photographic matches, Tardy et al. [51] found more precise and similar estimates between genetic-only, combined (genetic and photos), and corrected photographic-only matches than photographic-only matches, which were not corrected for the proportion of matched individuals to the total population abundance. Stevick et al. [52] further suggest that incorporating correction factors, related to photographic quality, may allow for the inclusion of lower-quality photographs to increase the dataset available for analysis with negligible bias. One of the main sources of error in photographic matching is the use of low-quality images [25], which are usually removed from many analyses, including capture-recapture studies [12]. In this study, based on focus and contrast alone (i.e., overall PQ), most photos used for matching were of excellent or good quality and allowed us to see subtle identifying features on the dorsal fin and body of the whales. When adding scores for angle and visibility for images that contained dorsal fins, 38% of them were classified as poor PQ. This low rating was due to loss of definition (i.e., focus) of photos taken from far away (the ship as opposed to the RHIB) and in photos that were cropped to isolate the dorsal fin. Studies that use the dorsal fin as the primary and sole identifying feature evaluate the dorsal fin angle to the camera and the amount of dorsal fin visible in the frame, with perpendicular or slightly angled and full display of the leading and trailing edges of the fin scoring the best values in contrast to oblique and obstructed views of the dorsal fin [11,16]. Interestingly, we found that the view of body marks, especially cookiecutter shark bite scars, were accentuated in oblique photos facilitating their use during the matching process. Therefore, maintaining angled photos that included the dorsal fin, and other parts of the body of the whales, contributed to their low scoring however maintaining them in the dataset was beneficial when evaluating the body of the whales as a whole.

An important source of error during photo-ID studies is the low or unknown stability of the identifying features [53]. Tezanos-Pinto et al. [54] argue that scars alone may not be reliably used to individually identify Bryde’s whales over long periods (8+ years) due to uncertainties about their stability. For minke whales, however, Bertulli et al. [26] found that the loss rate of cookiecutter shark bite scars was extremely low and by combining them with dorsal fin attributes, the rate of identification in the catalog was increased by more than a quarter. For Rice’s whales, cookiecutter shark bite scars were present on most whales and allowed whales with non-distinctive dorsal fins to be matched over time. Other body marks, such as nodules on the skin, other types of scars, nicks, and gaps on the peduncle also assisted in matching. Nicks and notches on the peduncle of other cetacean species have been used to reliably identify and match individuals, being used as primary and secondary features that allow poorly marked and challenging to photograph species to be studied using photo-ID methods (narwhals – *Monodon monoceros* – [55]; harbor porpoises – *Phocoena phocoena* – [56]). Although performing a quantitative analysis of the rate of loss and gain of identifying features in Rice’s whales was beyond the scope of this study, we found evidence that the persistence of dorsal fin attributes and body marks is high in the species. In whales matched by highly distinctive fins, including some confirmed by genetic matches, we noticed little to no change in the dorsal fin attributes and body marks used for identification for many years (e.g., nine years for catalog ID 2000 “Chip Tip”, Fig 7).

Lack of conspicuous identifying features, low photographic quality and unknown stability of marks are all elements that can lead to false-positive (considering two different individuals the same) or false-negative (considering one individual as two or more) matches, which in turn can bias population size estimates if the data are used to estimate abundance [12]. To construct the Rice’s whale photo-ID catalog, we evaluated and selected a large number of photos per individual and per sighting, including different sides and angles if available. We found this feasible due to the overall limited number of photographs and the small population size. By using multiple identification features per whale (i.e., combining dorsal fin attributes and body marks), we reduced potential matching errors, consistent with findings from other studies [e.g., 19,25,44] while at the same time maximizing the number of whales that could be individually identified and matched.

For Rice’s whales, the discovery curve showed an increase in the cumulative number of identified whales over time indicating that new individuals are still being added to the catalog. All except one whale was photographed inside the Core Area, despite surveys broadly covering the Gulf (Fig 1). The addition to the catalog of new, highly distinctive individuals photographed in 2019, suggests that whales may be moving in and out of the Core Area. Rice’s whales have been documented in other areas of the Gulf [9], and given the presence of suitable habitats throughout U.S. and Mexican waters [6,8], it is likely that new whales are yet to be captured in the photographic record. However, given the low acoustic detection rates in the western Gulf [9], we believe that the majority of the population is located in the eastern Gulf, including the Core Area.

Over the 15-year period of SEFSC surveys in the Gulf, indications of the presence of presumed mother and calf pairs during Rice’s whale sightings were rarely recorded (only 10 out of the total 161 sightings) with the first sighting in 2007. Only three adult females were identified as potential mothers, two of which are suspected to be multiparous, as they were observed with possibly two different calves each. Of the 25 sexed whales, 14 were females and adults (based on visually estimated size). Removing the three presumed mothers documented here, 11 other females could potentially be of reproductive age but have not been documented with an accompanying smaller whale.

The small number of potentially reproductive females and calves is concerning given the already small population size for Rice’s whales [4]. Growth and reproductive parameters for Rice’s whales are scarce. From stranding records, a 4.7 m female calf, a 6.9 m female juvenile, and a 12.65 m lactating whale were recorded in the Gulf [57]. For the closely related Bryde’s whale, length at sexual maturity is about 12 m and interbirth interval and age of weaning are 24 and 12 months, respectively, but females may not conceive every two years [58–61]. These stranding records indicate that young Rice’s whales and nursing mothers are present in the Gulf; however, without further monitoring of mother and calf pairs, establishing a relationship between size and sexual maturity, as well as reproduction rates is highly speculative. Catalog ID 20001 “Scyther” was photographed twice, four years apart, and on both occasions, accompanied by a smaller whale. Given the apparent small size of the presumed calf seen in the 2011 photograph, it was likely a different individual than the presumed calf seen in 2007. In 2016, catalog ID 6004 “Scoop” was photographed with a very small individual estimated at half her size. “Scoop” was photographed again in 2017, less than one year later, but no indications of a smaller whale nearby were noted; on this occasion “Scoop” was photographed and biopsied from the RHIB thereby increasing the chances of observers noticing a potential calf. Only in 2019, “Scoop” was re-sighted with a presumed calf, catalog ID 2001 “Scar”, which was roughly ⅔ of her size.

Genetic diversity is low for Rice’s whales [1]. As evidenced by the preliminary data from genetic analysis of the biopsy samples collected from the pair in 2007, catalog ID 20001 “Scyther” and the male presumed calf, it was not possible to accurately determine the extent to which the two whales were related based on microsatellite data (Wilcox Talbot, personal communication). Until robust genetic markers are further identified, morphological and behavioral descriptions observed from pairs of individuals and associations from long-term photo-ID data might be the sole data source in determining mother and calf relationships for Rice’s whales.

Many of the features used for individual identification during cetacean photo-ID studies, both in the dorsal fin and body of the animals, have confirmed or presumed anthropogenic origins. Amputations, linear cuts and marks, lacerations and deep triangular indentations especially in the head, dorsal and pectoral fins, and flukes of cetaceans are generally attributed to interactions with fishing gear [34,62–64]. Ten out of the 31 individually identified Rice’s whales presented clean-cut lacerations or notches on the dorsal fin. Four whales had linear lacerations that created amputations of the apex of their dorsal fins which were similar to amputations that were observed and attributed to fisheries interactions in cetaceans elsewhere [35,38,65] and included “loss of tissue, with partial or complete mutilation of the fin” [65]. Deep triangular and round notches were also present on the dorsal fin of Rice’s whales and similar notches have been attributed to fisheries interactions in multiple species of cetaceans, with triangular (or v-shaped) notches highly associated with fishing line interactions [35,38,66]. Preliminary skin lesion assessments of catalog ID 9000 “Lucky”, the whale with the large scar on the peduncle, identified healed linear impressions on its left fluke consistent with entanglement with fishing gear [67]. Soldevilla et al. [5] suggested that commercial bottom longline fisheries could pose a threat to Rice’s whales given the potential overlap between the fisheries’ sets and the whales’ foraging dives to the seafloor during daytime. However, the origin of these linear amputations and marks on Rice’s whales needs further investigation and other potential sources should also be considered, including recreational fisheries. To date, there are three documented interactions between Rice’s whales and fisheries gear, including fishing lines and a mortality associated with trap/pot entanglement [5].

Evidence of trauma was documented for two Rice’s whales that displayed large deformities on their bodies. Catalog ID 20001 “Scyther” had a laterally displaced lumbar vertebral dorsal spinous process first sighted in 2007. In 2011, muscle atrophy and decreasing body condition made this deformity more readily visible; this disarticulation could have been caused by high-impact blunt force trauma or rotational injury (Ewing, personal communication). Catalog ID 9000 “Lucky” presented a large-healed scar on the peduncle, indicative of a vessel strike. Similar injuries, typically consisting of linear or slightly curved lacerations [64], have been reported for multiple species of cetaceans and attributed to vessel and/or propeller strikes [37,66,68]. Worldwide, large whales are at high risk of vessel strikes given the overlap between species’ distribution ranges and large vessel activity [69]. Soldevilla et al. [5] evaluated vessel strike risk for Rice’s whales in the Core Area using automatic identification systems (AIS) data for large commercial vessels. Although vessel traffic was considered relatively low compared to the northwestern Gulf, these whales are at risk of collision given their nocturnal behavior of staying at the water’s surface for 88% of the time, within the reach of the draft of these vessels. To date, one Rice’s whale mortality is attributed to a commercial vessel that hit a lactating female, which remained wrapped around the bow of the vessel after the strike and was examined [57]. The opportunity to examine Rice’s whale carcasses is extremely rare given the offshore nature of their distribution, therefore evidence of ship strikes and other mortalities is severely underestimated. In an enclosed gulf of New Zealand where dead Bryde’s whales can be recovered for examination, 85% of the known causes of mortality were attributed to vessel strikes [70]. Furthermore, from tag data, it was determined that these whales spent 91% of their nighttime at the surface, a similar behavior documented for Rice’s whales in the Gulf [5].

The risk of strikes from recreational fishing vessels also needs to be assessed. Given the relatively short distance of roughly 50–60 nautical miles between the Rice’s whale Core Area and the U.S. shore, recreational fishing vessels access these waters somewhat frequently. During the 2019 survey (GU1901), multiple fishing vessels, usually less than 20 m (65 feet) in length, were seen within the Core Area (Aichinger Dias, personal observation). In the U.S., AIS are not required from vessels less than 20 m in length; therefore, they are grossly underrepresented when assessing the risk of vessel strikes to whales [5,71]. Furthermore, recreational small vessels (<15 m in length) are responsible for large accounts of whale strikes, and are more likely to kill smaller individuals, like calves and juveniles, than adults [72].

In addition to fisheries and vessel strike risks, Rice’s whales are also subjected to exposure to marine debris. Plastic has been identified as a major threat to cetaceans, causing entanglements, death by ingestion [73,74], and suppression of their immune system [75]. Catalog ID 7003 “Witch Hazel” was first photographed during a survey in November 2018 with a sunken dorsum, indicative of loss of muscle mass and poor body condition (Fig 13). When this whale stranded in January 2019 a piece of hard plastic was found in the stomach and may have caused acute gastric necrosis and hemorrhage, contributing to inanition and subsequent death (Boyd, personal communication).

## Conclusion

This study confirms that photo-ID techniques can be applied to Rice’s whales to identify individuals across multiple years. Whales were individually identified by dorsal fin attributes and body marks, used in combination whenever possible. We believe the photographic record gathered here is the beginning of a dataset that can be used to fill major gaps in understanding Rice’s whale ecology. Our study identified 31 individuals of a current population size estimated at 51 whales [4]. However, the confidence interval of this estimation is wide, ranging from 20 to 130 animals, reflecting the need for more accurate population size estimates.

Outside of the Core Area, Rice’s whales have been documented in the northcentral and northwestern Gulf [2,8,9]. Whereas in Mexican waters, the presence of suitable habitat coupled with acoustic detections support additional areas of use [6,9]. Nevertheless, Rice’s whale distribution and connectivity across areas are poorly understood. Long-term systematic photo-ID surveys would allow us to better document whale movement and potential exposure to anthropogenic impacts. The photographs gathered here suggest that Rice’s whales may be regularly exposed to vessel strikes and fisheries interactions. These interactions could happen outside the Core Area and beyond U.S. waters; however, without seasonal and inter-annual long-term distribution and movement data, we cannot isolate high risk zones.

Our study also documented mother and calf pairs with only three females identified as presumed mothers. The rarity of observations of mother and calf pairs and the relative low number of reproductive females are concerning especially considering the already small population size. Further photo-ID survey efforts would allow us to not only monitor the already documented pairs but also gather data to estimate rates of reproduction and survivorship.

**Fig 14:**
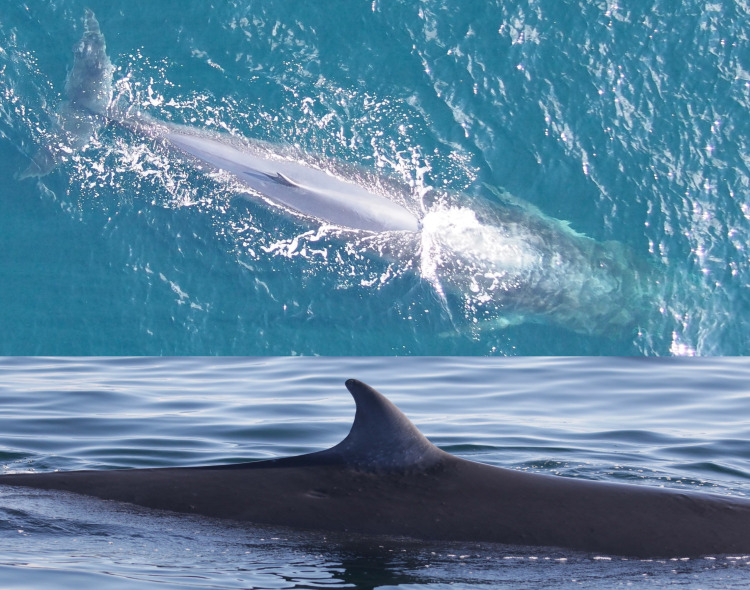
Catalog ID 20023 “Edna”, photographed with a UAV (top) and matched to the vessel-based photograph by a cookiecutter shark bite scar and scars on the dorsum (bottom).

Finally, with the advancement of Unmanned Aerial Vehicles (UAVs) and their use in cetacean photo-ID [76,77] and visual health assessments [78], we believe UAVs are an important tool in the study of Rice’s whales. A limited number of aerial photographs are available for Rice’s whales and we successfully matched catalog ID 20023 “Edna” between vessel-based and UAV imagery using a cookiecutter shark bite scar and another scar on the dorsum (Fig 14). We believe that combining and adapting photo-ID methods with new technologies will provide a better understanding of Rice’s whale ecology and health needed to preserve this already critically endangered species.

## Supporting information

S1 File Photographic quality assessment. Document describing photographic scoring parameters based on focus, contrast, dorsal fin angle and visibility. Resulting overall and dorsal fin photo quality (PQ) are also exemplified.
(DOCX)

S2 File Sighting history, location and sex (if known) for all individually identified and/or sampled Rice’s whales. Mother and calf pairs are indicated by their respective catalog ID numbers followed by year seen together.
(XLSX)

S3 File Microsatellite genotypes for all sampled Rice’s whales included in the photo-ID catalog. The genetic data was obtained by [1,2].
(XLSX)
