# Supplementary material for: Biological findings from a newly developed photo-identification catalog for the critically endangered Rice’s whale (Balaenoptera ricei)
Source: PLoS One. 2025 Sep 8;20(9):e0331010. doi: 10.1371/journal.pone.0331010 (PMC12416752; doi:10.1371/journal.pone.0331010)
Supplement: S1 File — Document describing photographic scoring parameters based on focus, contrast, dorsal fin angle and visibility. Resulting overall and dorsal fin photo quality (PQ) are also exemplified. (DOCX) [file pone.0331010.s001.docx]

Photographic quality assessment used for: Biological findings from a newly developed photo-identification catalog for the critically endangered Rice’s whale (*Balaenoptera ricei*)

Supporting documentation describing photographic scoring parameters based on focus, contrast, dorsal fin angle and visibility used for the development of the Rice’s whale photo-identification catalog. Resulting overall and dorsal fin photo quality (PQ) are also exemplified.

# Focus/clarity: crispness or sharpness of the image. Lack of clarity may be caused by poor focus, excessive enlargement (large pixels), and motion resulting in blurry images (adapted from [1, 2]). Dorsal fin images cropped from larger photos were rated independently from the original image.

Excellent = 1: small features are visible.


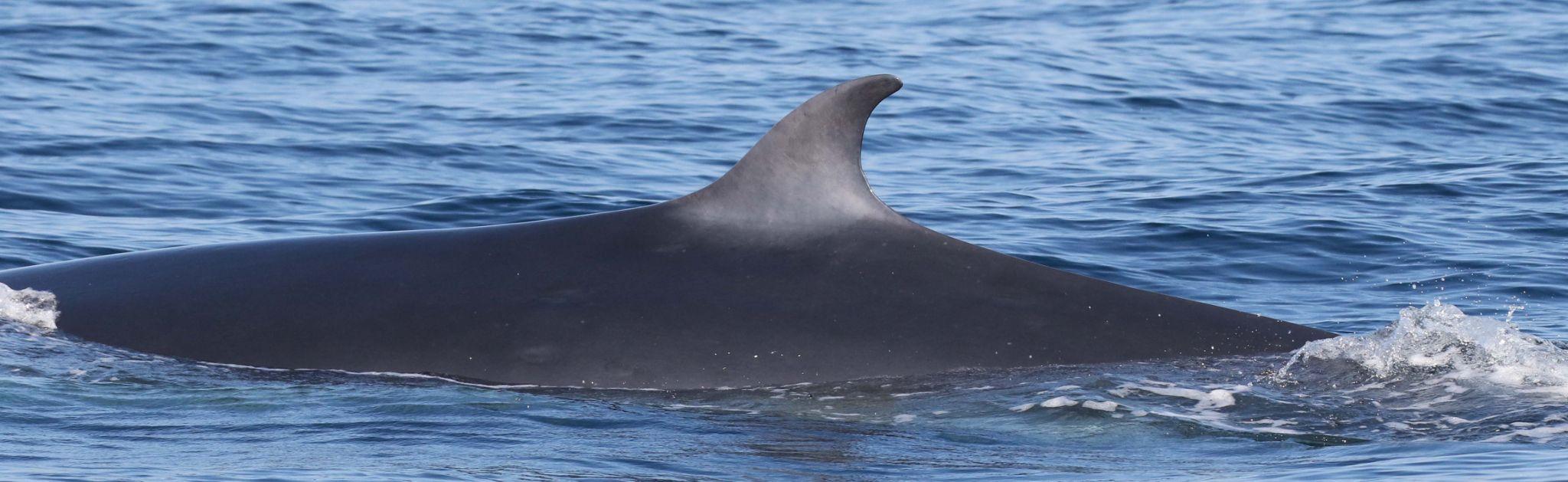


Moderate/reasonable = 4: some loss of definition but larger features are still visible.


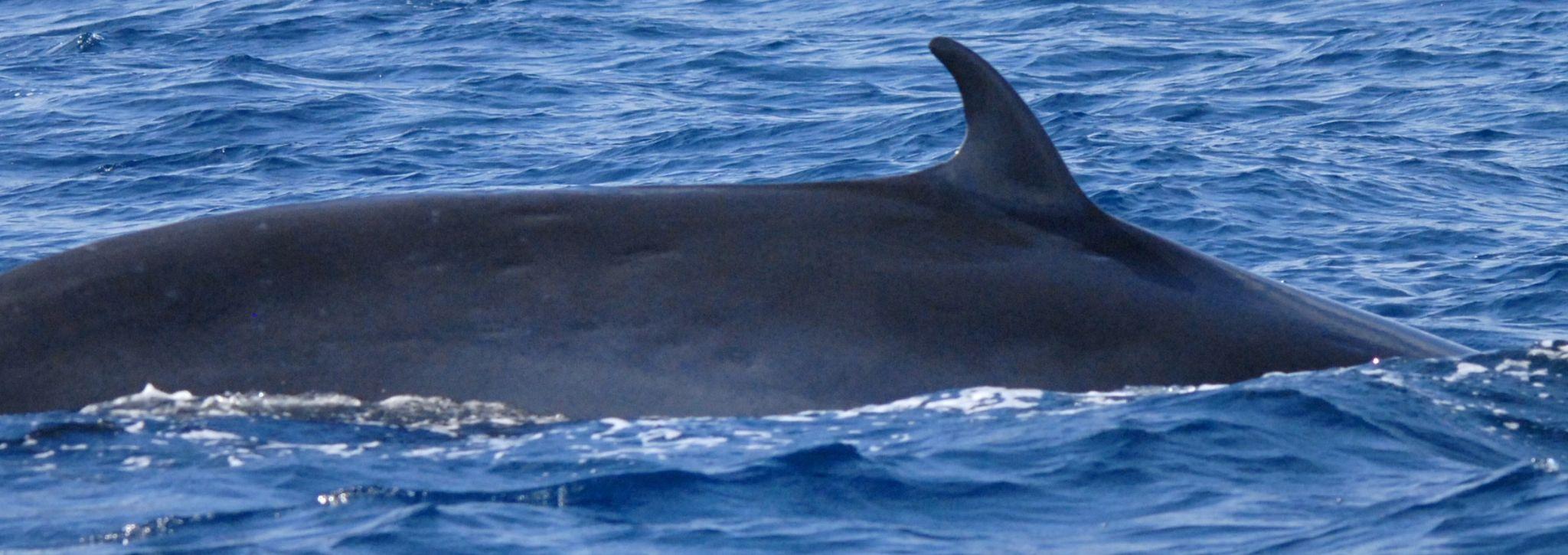


Poor = 9: blurry and features hardly seen (if at all).


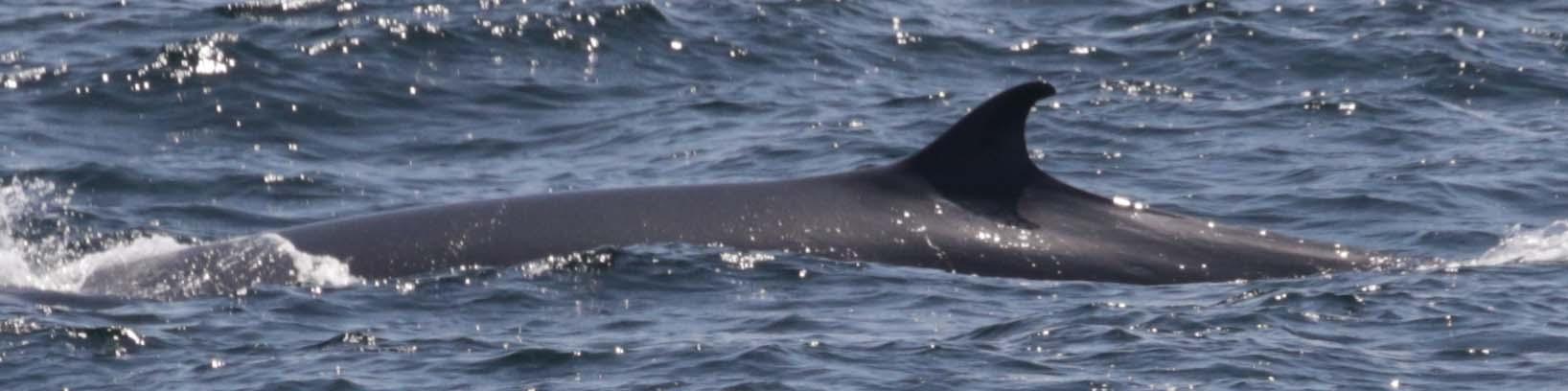


# Contrast/exposure: range of tones in the image. Features lack definition and may be difficult to see in images with excessive (overexposed, too light) or minimal contrast (underexposed, too dark) [1].

Ideal = 1: typical coloration of the whale visible; chevron (if present) is visible. No over or under exposure and all details and outlines are visible [2].


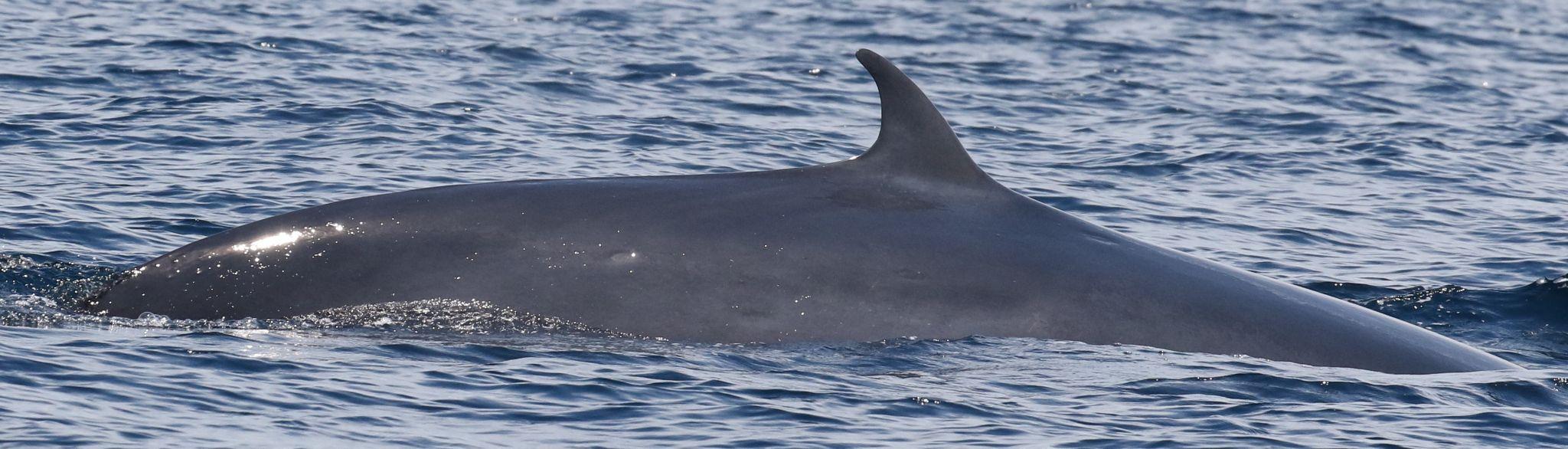


Reasonable = 3: little light or dark but most details are clearly seen (adapted [2]).


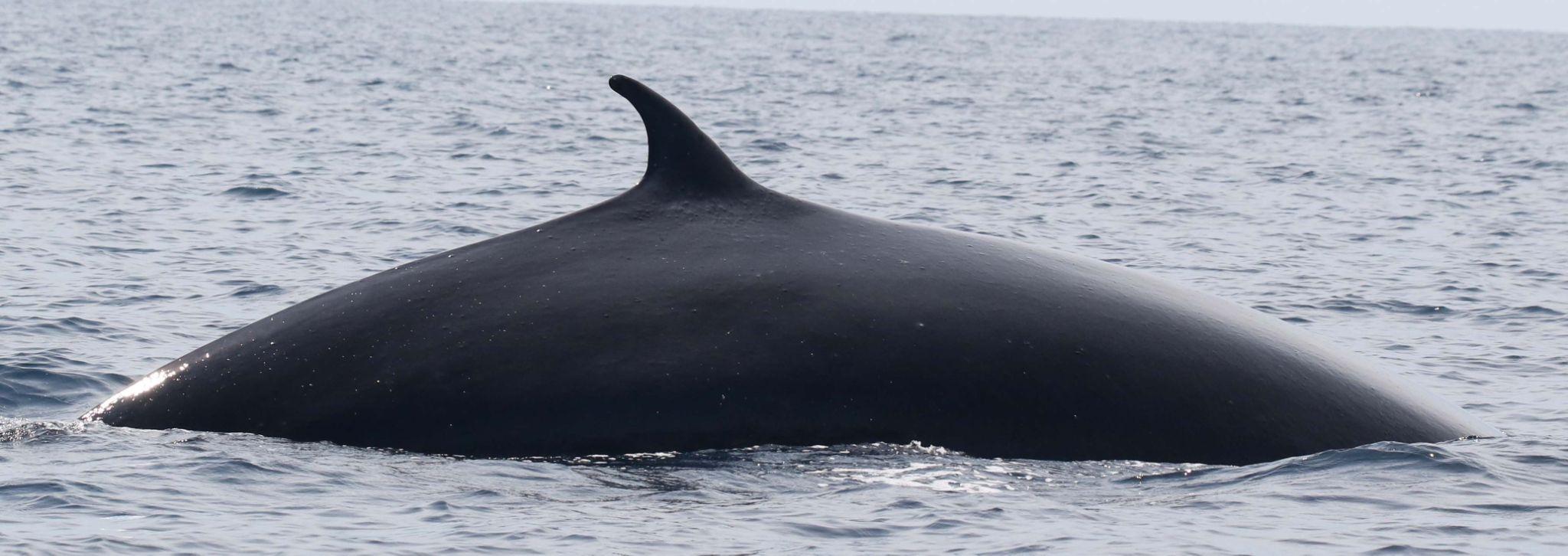


Poor = 9: under or over-exposed, only some details are seen (adapted from [2]); whale appears mostly uniformly dark or too light.


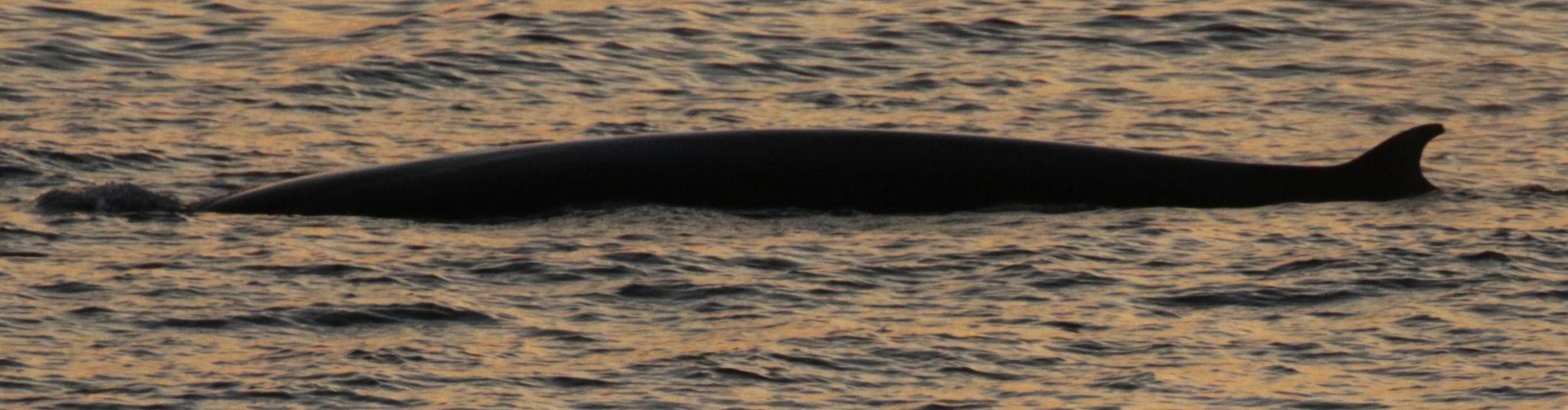


#

# Angle: angle of the dorsal fin to the camera [1].

Perpendicular = 1: The whale is 80-90° to the camera/photographer [3]. Features in the leading and/or trailing edge are fully visible.


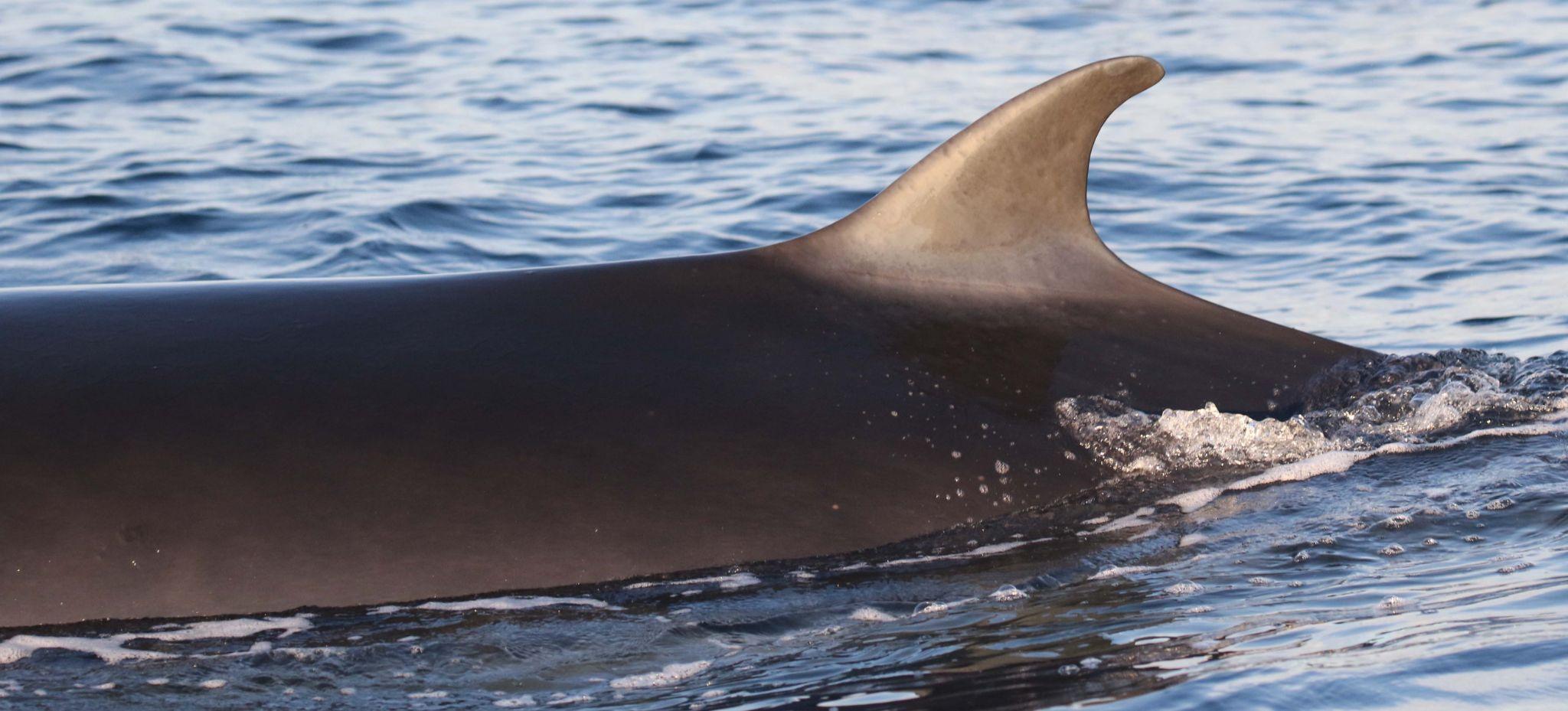


Slight = 2: The whale is 40-80° to the camera/photographer (adapted from [3]).

Features in the leading and/or trailing edge are still visible.


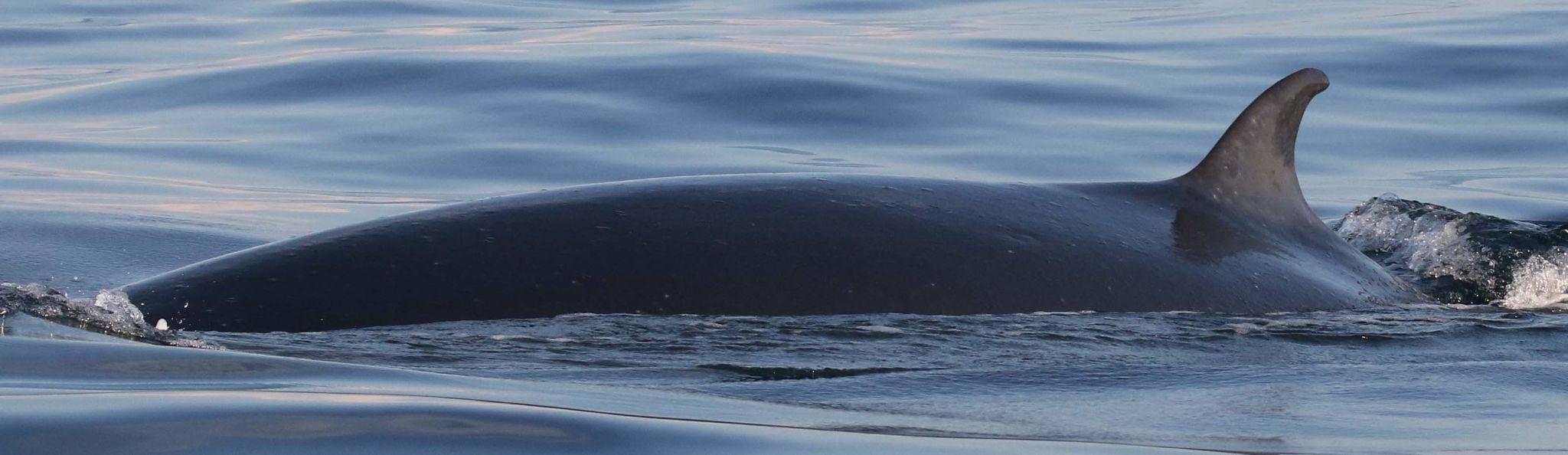


Oblique = 9: The whale is 0-40° to the camera; whales may be headed toward or away from the photographer [3].


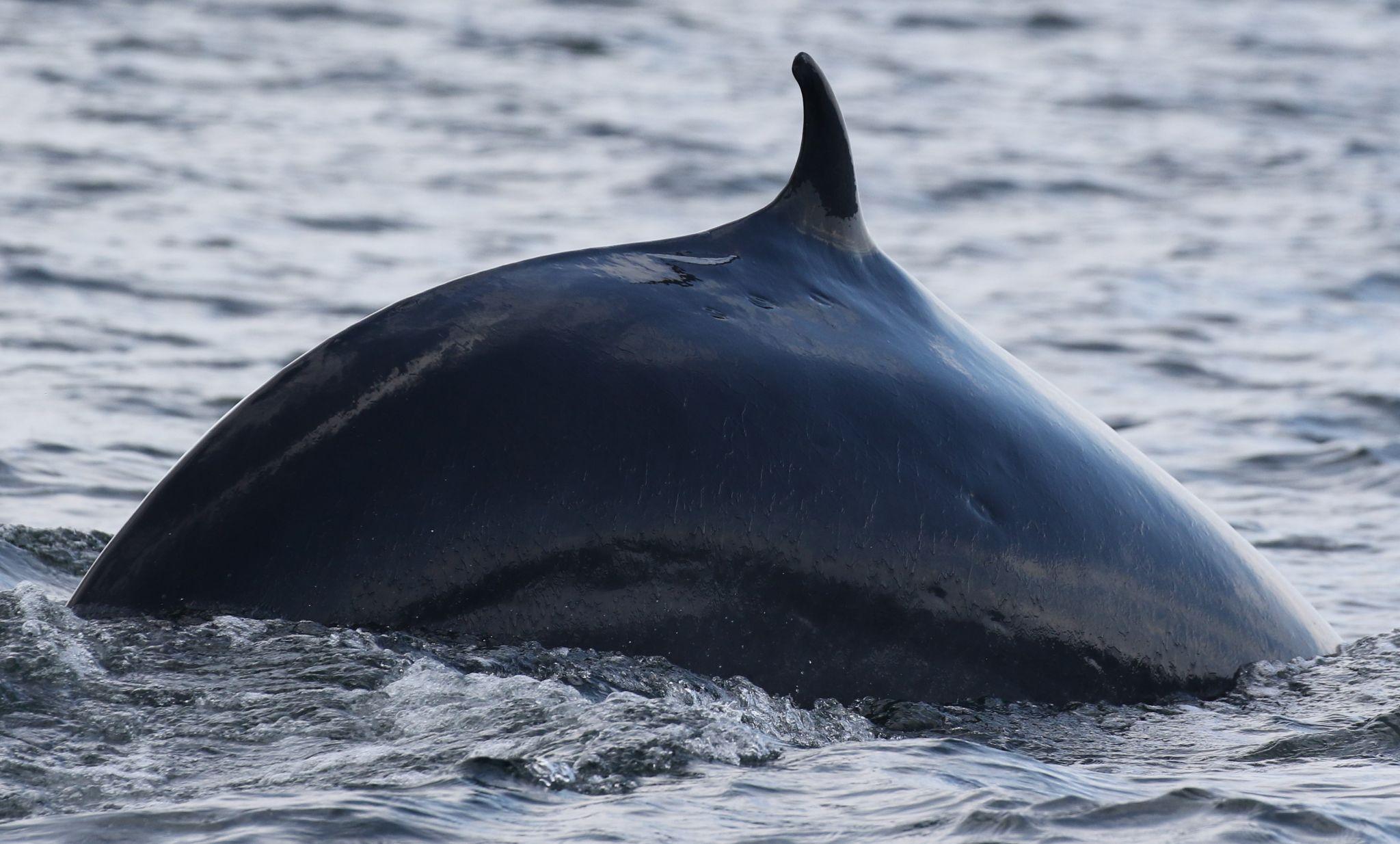


Not visible = 100: the dorsal fin is not present in the image.


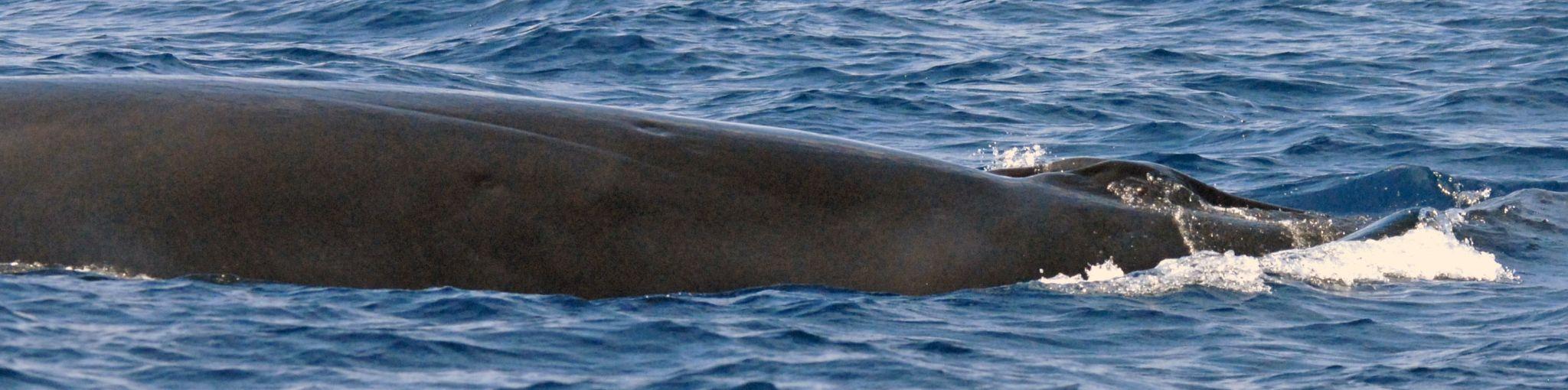


#

# Visibility: if the dorsal fin is fully or partially visible in the image; obstructions may be caused by water, other individuals, etc [1].

Fully visible = 1: leading and trailing edges of the dorsal fin are fully visible [2].


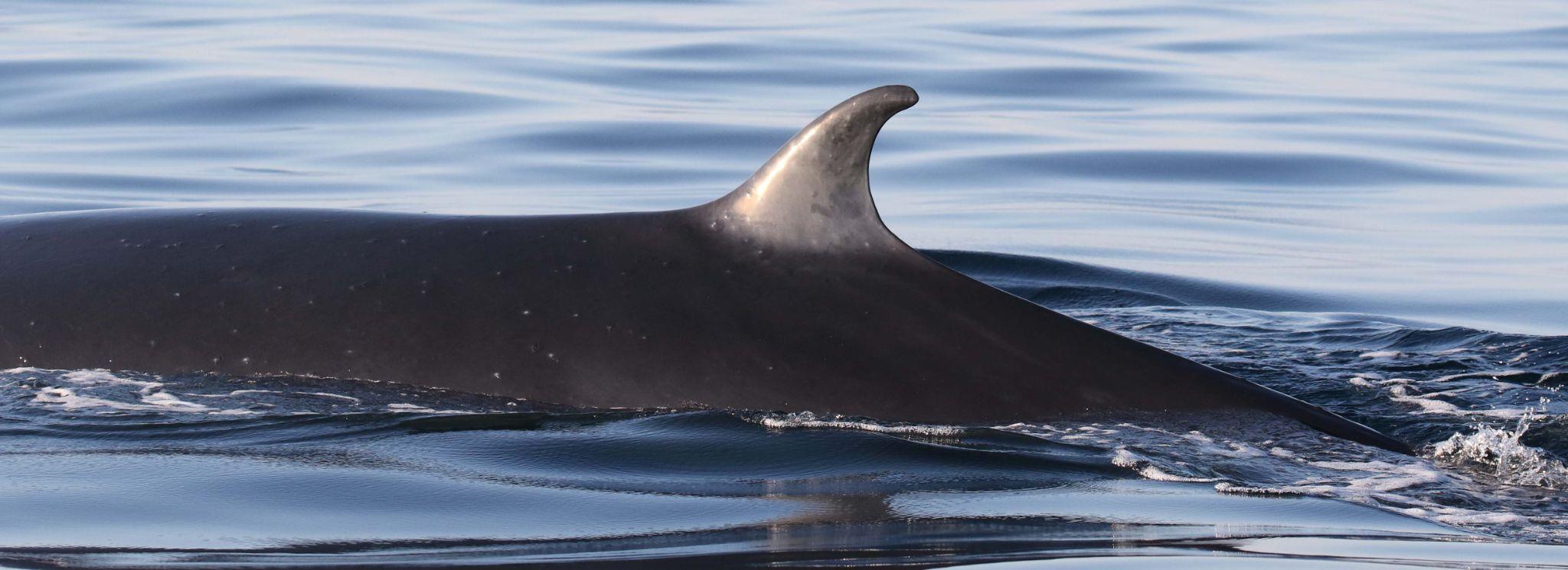


Partially obscured = 8: leading and trailing edges of the dorsal fin are partially obscured [2].


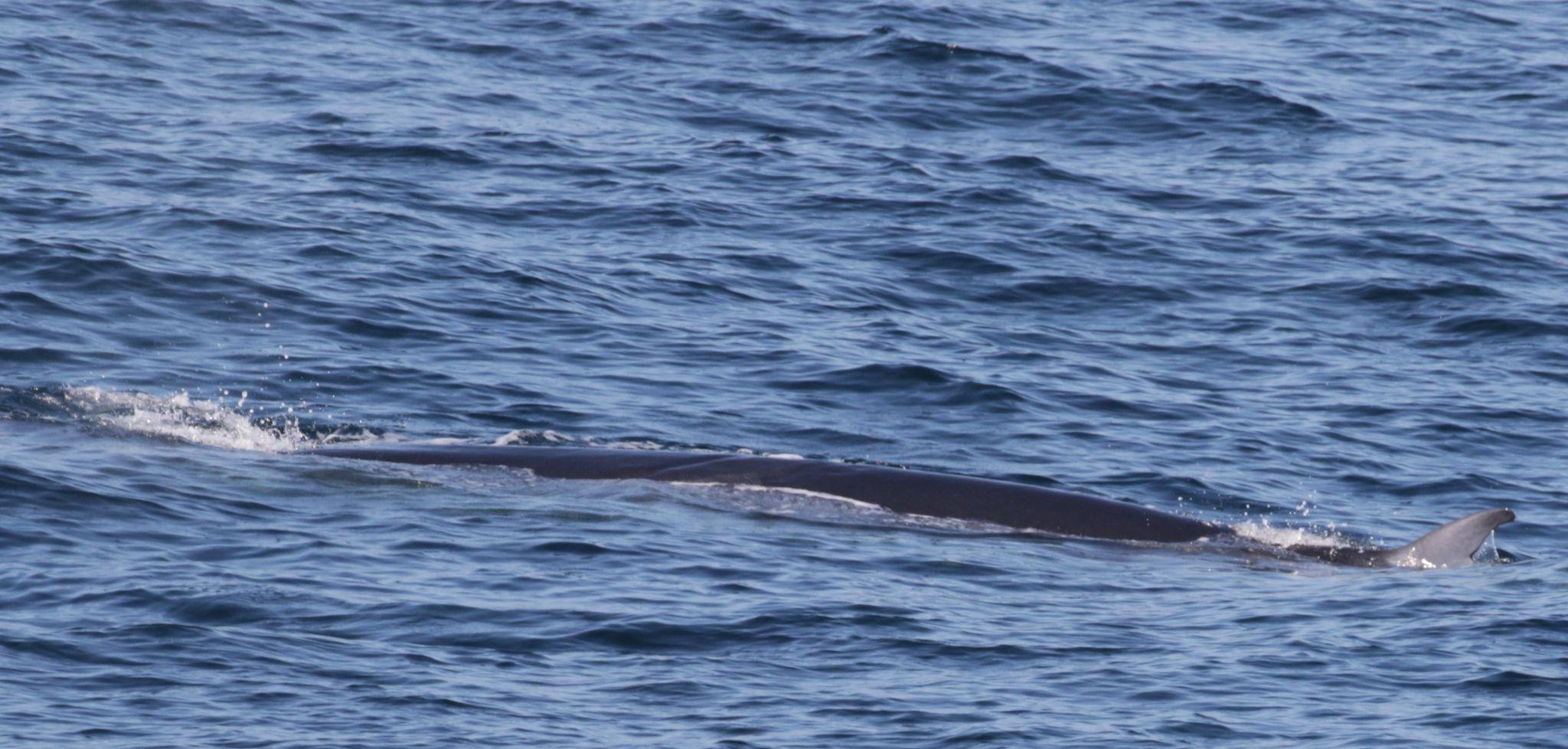


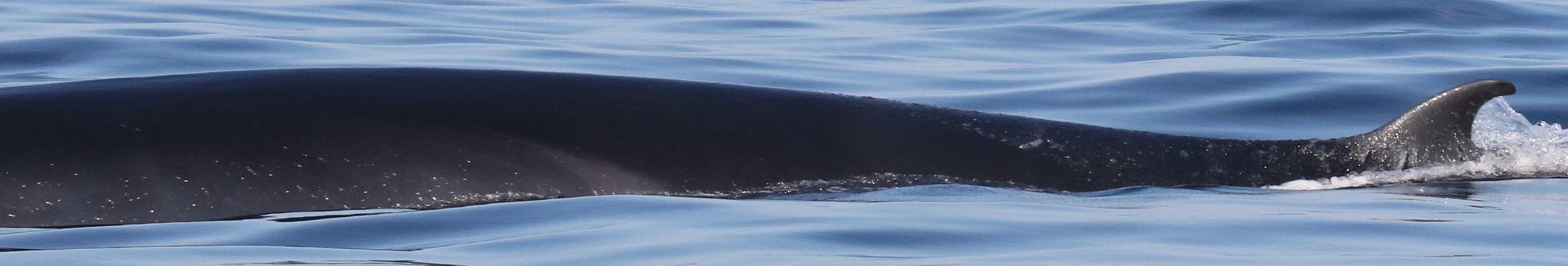


Not visible = 100: the dorsal fin is not present in the image.


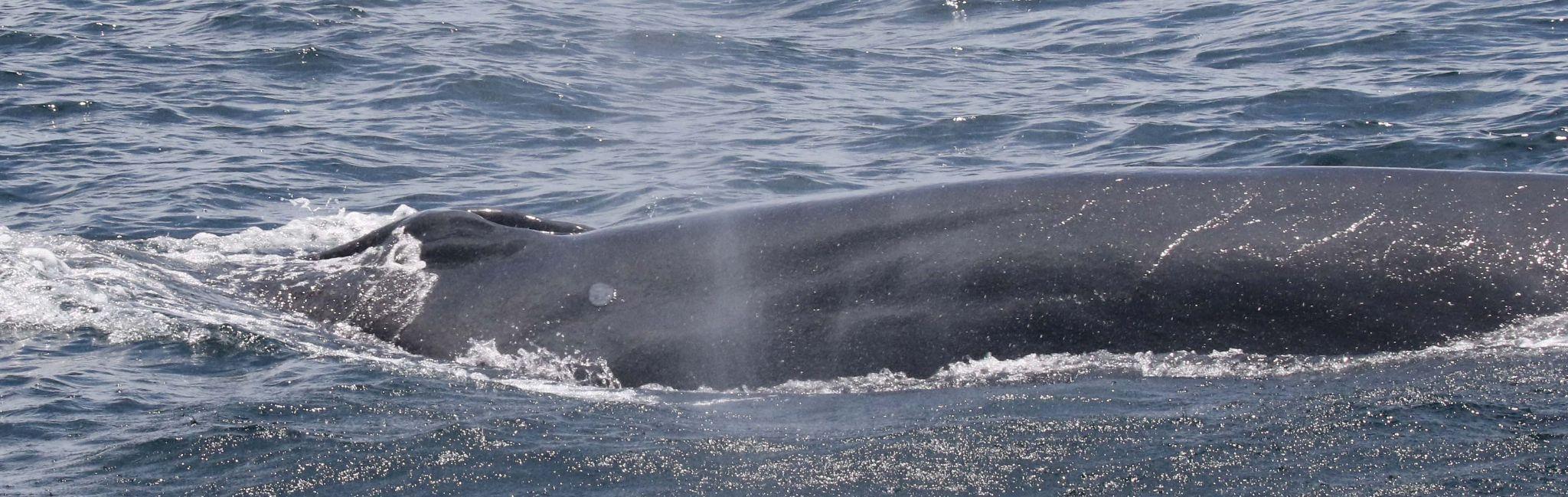


#

# Overall PQ: evaluated for all images; sum of focus and contrast.

| **Low** | **Upper** | **PQ** |
| --- | --- | --- |
| 2 | 4 | Excellent |
| 5 | 7 | Good |
| 10 | 10 | Fair |
| 11 |  | Poor |

# Dorsal fin PQ: evaluated for images containing dorsal fins; sum of focus, contrast, angle and visibility.

| **Low** | **Upper** | **DF_PQ** |
| --- | --- | --- |
| 4 | 6 | Excellent |
| 7 | 9 | Good |
| 10 | 11 | Fair |
| 12 | 35 | Poor |
| 100+ |  | NA (dorsal fin not visible) |

#

# PQ examples

PQ: excellent (2)

DF_PQ: excellent (4)


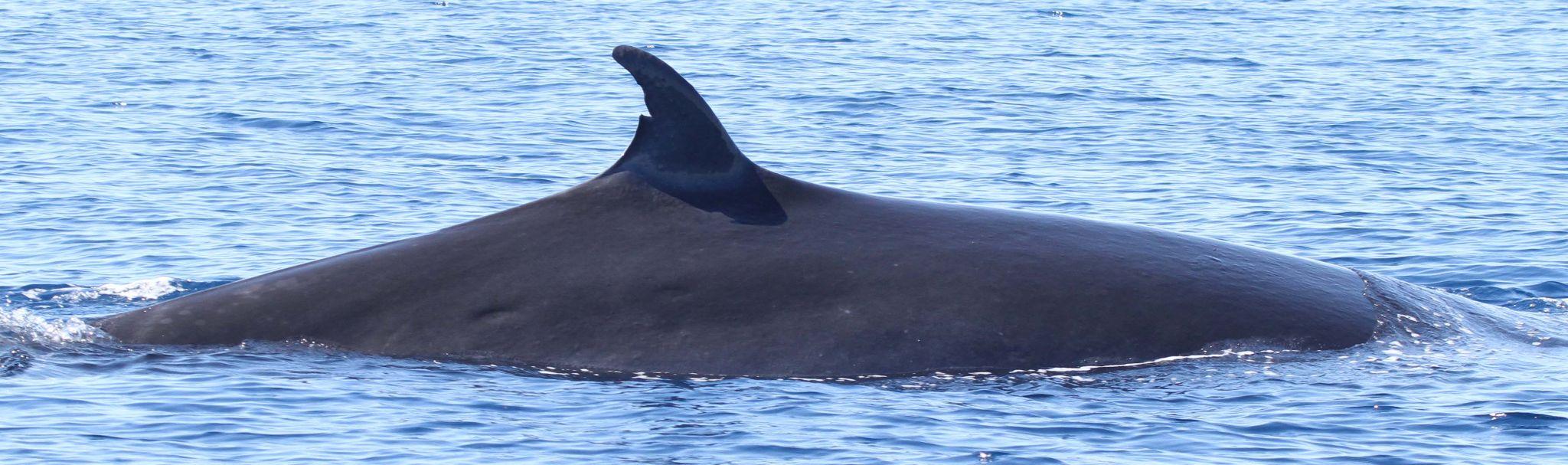


PQ: excellent (2)

DF_PQ: poor (12)


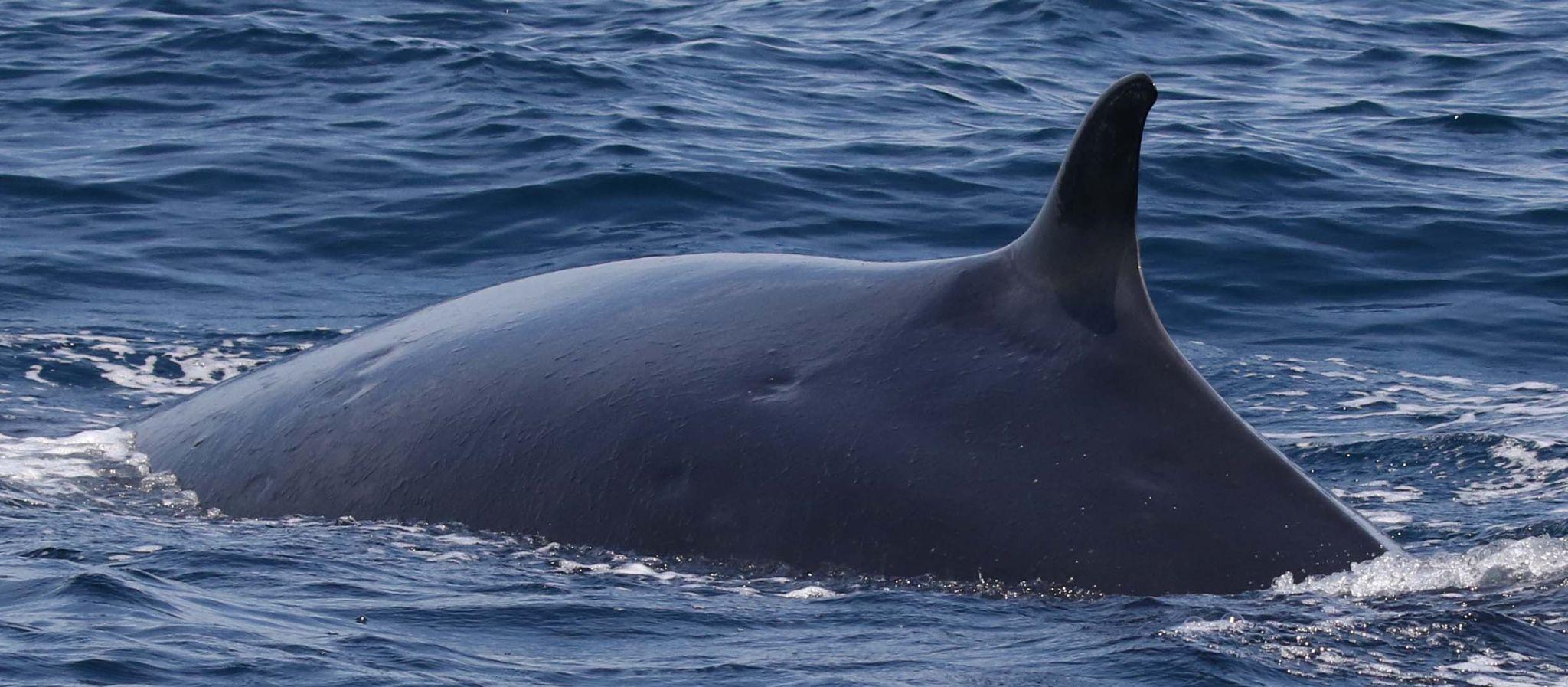


PQ: good (5)

DF_PQ: good (8)


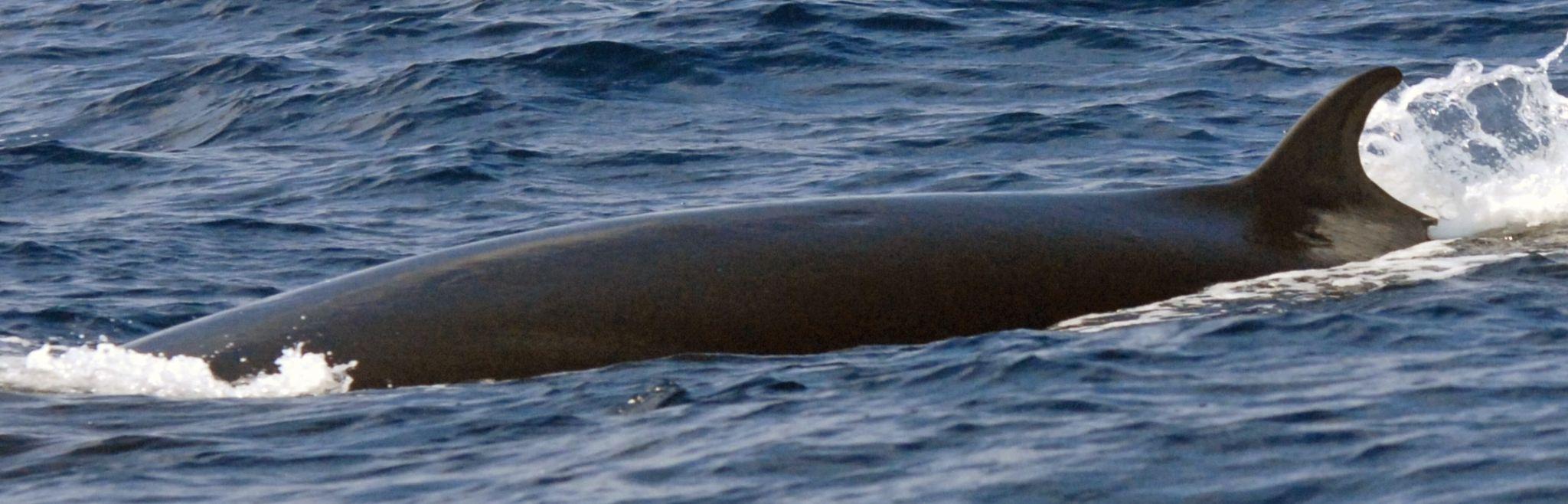


PQ: fair (10)

DF_PQ: poor (27)


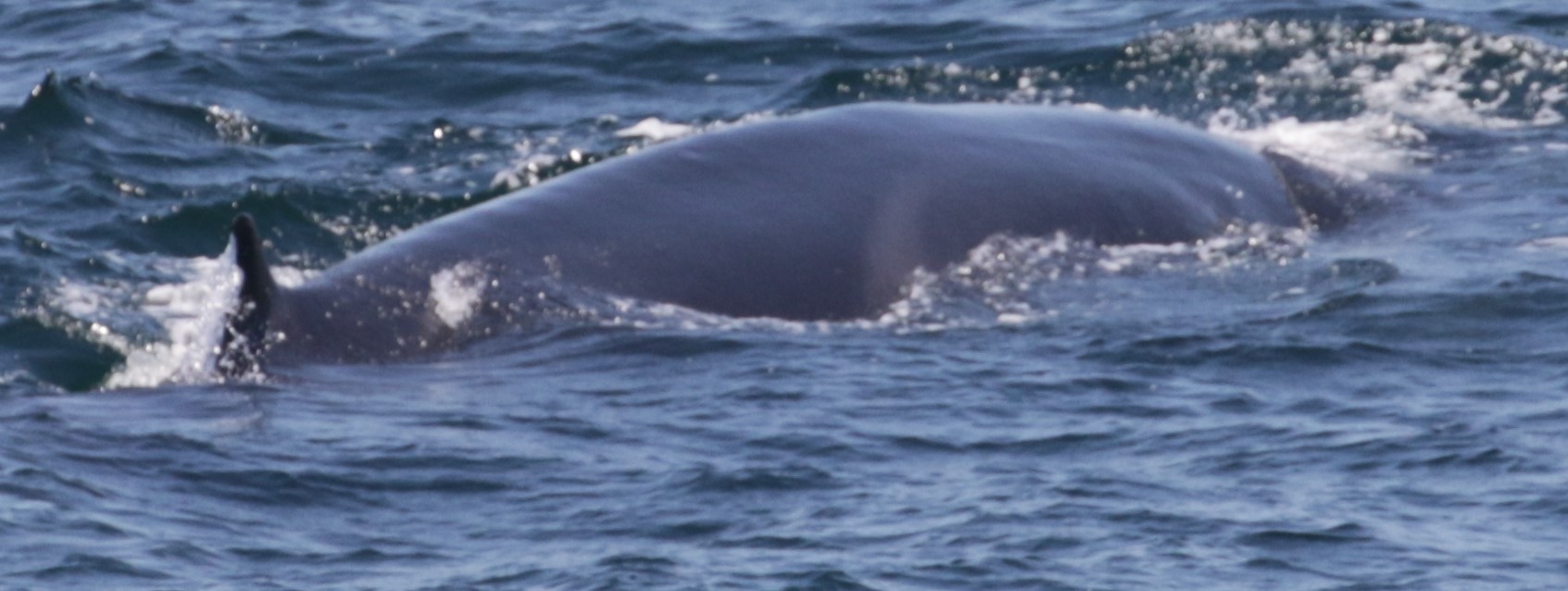


PQ: poor (12)

DF_PQ: poor (22)


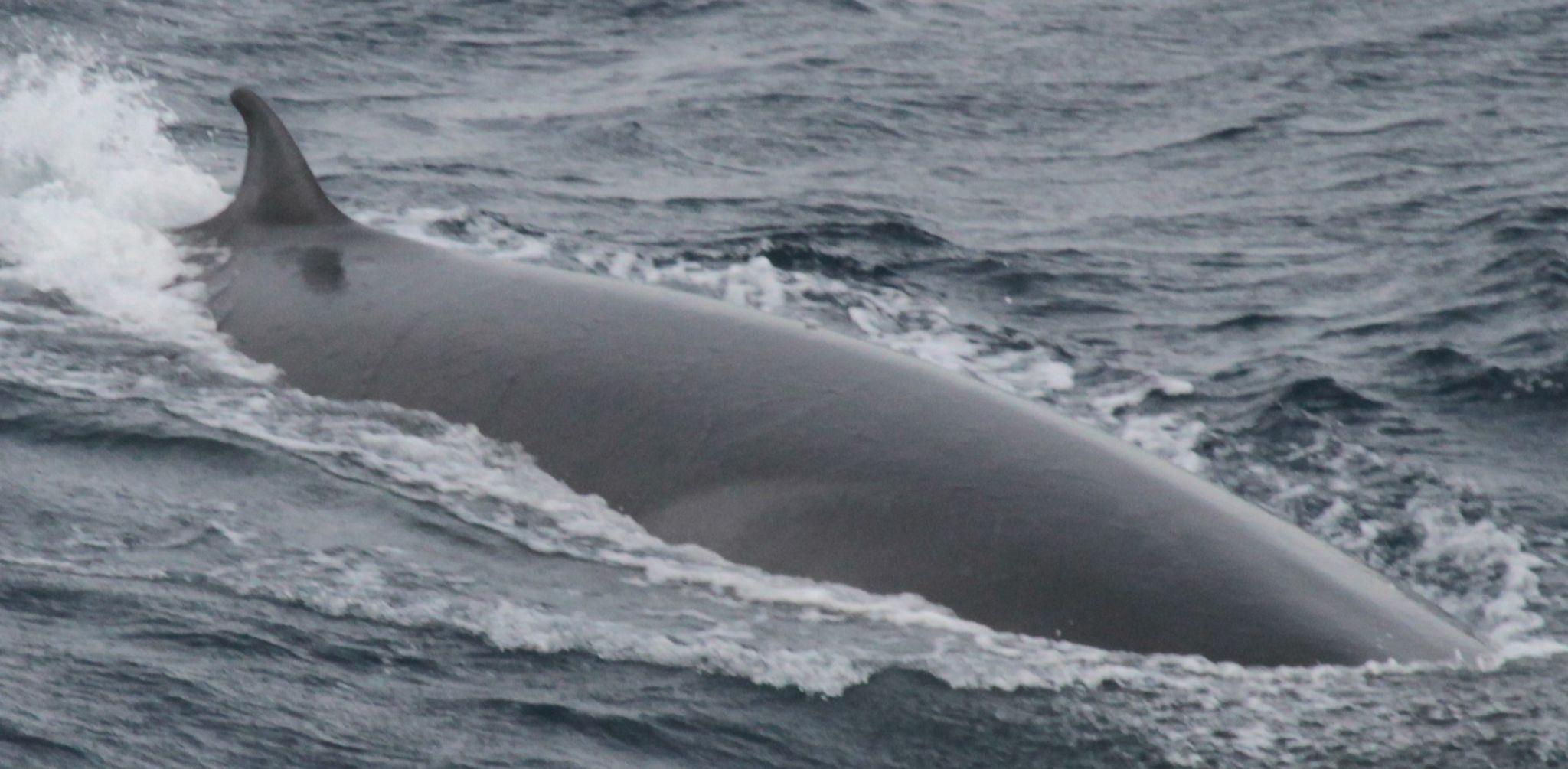


# References

1. Rosel P, Mullin K, Garrison L, Schwacke L, Adams J, Balmer B, et al. Photo-identification capture-mark-recapture techniques for estimating abundance of bay, sound and estuary populations of bottlenose dolphins along the US East coast and Gulf of Mexico: A Workshop Report. NOAA Technical Memorandum NMFS-SEFSC-621. 2011, 30p.

2. Hupman KE, Pawley MD, Lea C, Grimes C, Voswinkel S, Roe WD, et al. Viability of photo-identification as a tool to examine the prevalence of lesions on free-ranging common dolphins (*Delphinus* sp.). Aquatic Mammals. 2017;43(3):264-78. doi: 10.1578/am.43.3.2017.264.

3. Olson P, Galletti Vernazzani B, Torres Florez J. Southern Hemisphere blue whale catalogue photo quality coding guide. International Whaling Commission SC/68C/PH/02. 2021.
